# PP2A licenses the FANCD2/FANCI complex for chromosome loading

**DOI:** 10.1016/j.celrep.2024.114971

**Published:** 2024-11-12

**Authors:** Di Yang, Fengxiang Bai, David Lopez Martinez, Hannan Xu, Ai Johjima-Murata, Lily Jiaqi Cao, Martin A. Cohn

**Affiliations:** 1 Department of Biochemistry, University of Oxford, Oxford OX1 3QU, UK

**Keywords:** PP2A, FANCD2, FANCD2/FANCI complex, Fanconi anemia, genome stability, dephosphorylation, DNA interstrand crosslink repair, ICL repair, phosphorylation

## Abstract

The Fanconi anemia (FA) pathway removes interstrand crosslinks (ICLs) between the Watson-Crick strands of the DNA double helix in humans. Central to the pathway is the FANCD2/FANCI complex, which must be loaded onto chromosomes. Here, we report the identification of a PP2A phosphatase complex, which specifically dephosphorylates an inhibitory cluster in FANCD2, thereby licensing its loading in response to DNA damage. We show that PP2A is required for normal monoubiquitination of the FANCD2/FANCI complex and for its loading onto chromosomes. We have fully reconstituted a coupled dephosphorylation-ubiquitination reaction *in vitro* using a highly purified PP2A complex. Using super-resolution live-cell single-molecule tracking, we show how PP2A switches on the FA pathway in response to ICLs and that cells are sensitive to ICL-forming drugs in the absence of PP2A. Our work uncovers a mechanism where PP2A facilitates the activation of the FA pathway by licensing chromosome loading of the FANCD2/FANCI complex.

## Highlights

• The PP2A protein phosphatase licenses the FANCD2/FANCI complex to be loaded onto chromosomes
• PP2A promotes DNA interstrand crosslink repair in humans
• Activation of the Fanconi anemia pathway depends on PP2A dephosphorylation of FANCD2
• Sensitizing cancer cells by perturbing PP2A could provide clinical therapeutic opportunities

## Graphical abstract

**Figure:**
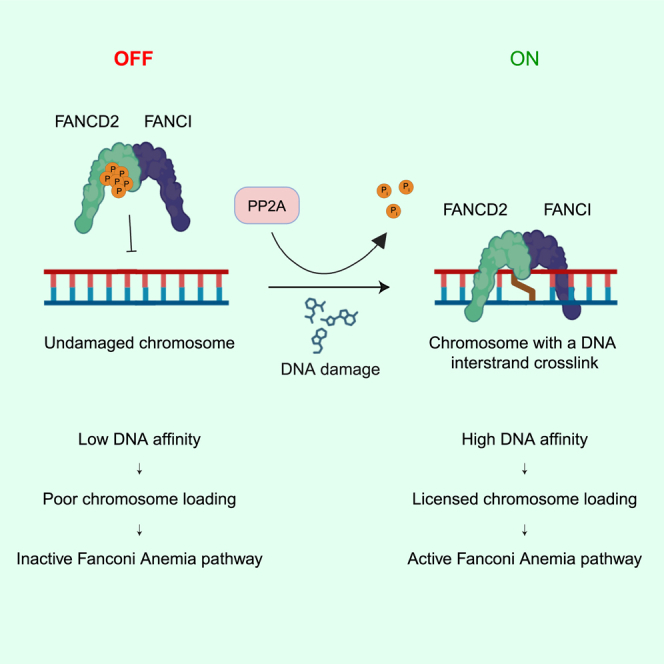

## Teaser

Yang et al. demonstrate how the PP2A protein phosphatase complex specifically dephosphorylates FANCD2, thereby licensing the FANCD2/FANCI complex to be loaded onto chromosomes for DNA interstrand crosslink repair. Suppression of PP2A activity deregulates the Fanconi anemia pathway and sensitizes cells to DNA interstrand crosslinks.

## Introduction

Maintenance of the human genome during cell division is essential for our health. As our genomes experience damage from a variety of factors of both endogenous and exogenous sources, cells have evolved multiple intricate pathways to sense and repair different types of DNA damage. A particularly toxic type of DNA damage is the covalent interstrand crosslinks (ICLs) between the Watson-Crick strands of the double helix in our chromosomes. Such ICLs will prevent the proper separation of the 2 strands, thereby affecting both transcription and replication, and in turn jeopardizing the fidelity of chromosome segregation during mitosis.

Perhaps not surprisingly, failure to repair ICLs causes pathology in humans and is the underlying cause of a rare autosomal recessive disease called Fanconi anemia (FA). FA affects approximately 1 in 200,000–400,000 individuals in the general population but has a higher incidence in some ethnic groups. FA patients are typically born with congenital abnormalities and develop bone marrow failure at an early age.^1^ Patients are also at an increased risk of developing various types of cancers, especially acute myelogenous leukemia, but also head and neck cancers, typically in adult age.

The underlying pathway that is dysfunctional in FA patients is the FA DNA repair pathway.^2^ Currently, 23 different FA proteins are known, and mutation of any one of the corresponding genes can cause the disease. Together with a number of other cellular DNA repair factors, the FA proteins coordinate a complex repair process, entailing nucleotide excision repair, translesion synthesis, and homologous recombination. Central to the pathway are 2 FA proteins forming the heterodimeric FANCD2/FANCI complex. At an early stage in the pathway, this complex must be loaded onto the chromosomes. Once loaded, both subunits can be monoubiquitinated,^3^ which results in a conformational change in the complex, now adapting a more closed conformation that encircles the DNA.^4^ In its monoubiquitinated circular form, the complex is activated, and the FA pathway can progress. Failure of the complex to load onto DNA prevents monoubiquitination and completely inactivates the pathway. Therefore, the step of chromosome loading defines a key regulatory step of the pathway. However, the mechanism regulating this step is poorly understood.

Previous work uncovered how phosphorylation of the FANCD2/FANCI complex by ataxia-telangiectasia mutated and Rad3-related (ATR) is required for its monoubiquitination in response to ICLs in living cells.^5^^,^^6^^,^^7^^,^^8^^,^^9^ At the same time, phosphorylation of a cluster of 6 other amino acids closely located at 882–898 in FANCD2 was shown to completely prevent loading of the complex in the absence of DNA damage.^10^ Given the strong inhibition of loading by phosphorylation of this cluster, and therefore effective suppression of the FA pathway, we speculated that an unknown phosphatase activity could exist, which could reverse the inhibition and potentially license the complex for loading onto chromosomes in response to DNA damage.

Here, we report the identification of the PP2A phosphatase complex (PPP2CA/PPP2R1A/PPP2R3A) as a specific enzyme dephosphorylating the inhibitory cluster in FANCD2, thereby licensing the FANCD2/FANCI complex for loading onto chromosomes, permitting its monoubiquitination, and activating the FA pathway. Inhibiting cellular PP2A activity reduces its monoubiquitination, prevents loading of the FANCD2/FANCI complex, and suppresses activation of the pathway. Mechanistically, full reconstitution of the coupled dephosphorylation-monoubiquitination reactions *in vitro* demonstrates a direct dephosphorylation of FANCD2, thereby permitting monoubiquitination. Ensemble and super-resolution single-molecule live-cell imaging demonstrate how PP2A is required for genome-wide loading of the FANCD2/FANCI complex in response to ICLs. Taken together, the findings explain a key activation step for the FANCD2/FANCI complex and for the FA pathway.

## Results

### PP2A activity is required for normal FANCD2 monoubiquitination

**Figure 1:**
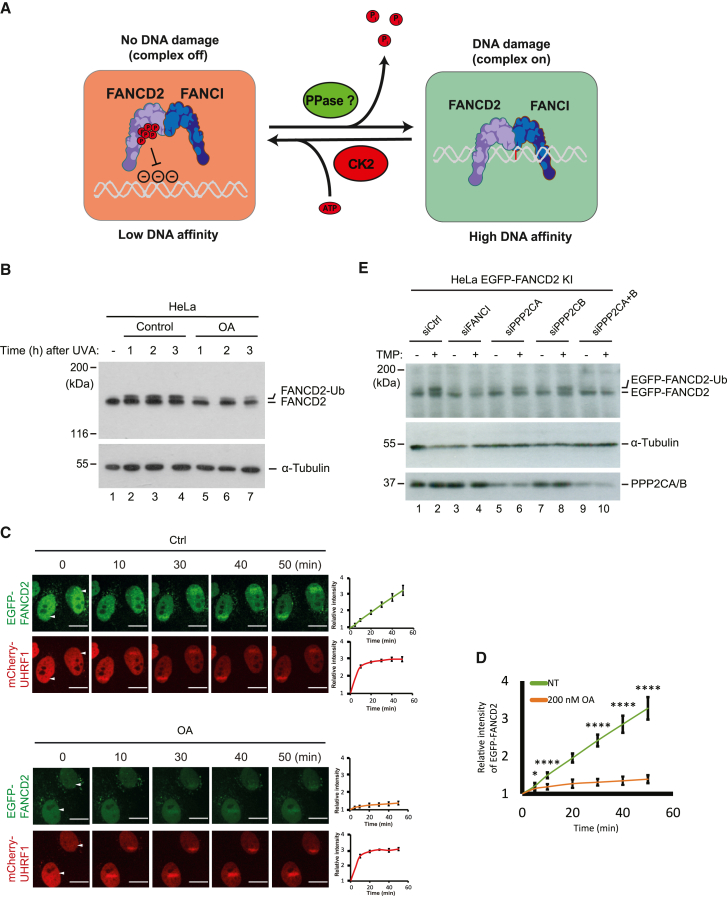
FANCD2 is dephosphorylated by a phosphatase belonging to the PP2A family in response to ICLs (A) Schematic representation of the hypothesis that FANCD2 needs to be dephosphorylated by an unknown phosphatase for it to be relieved from the inhibitory phosphorylation mediated by CK2. Phosphorylation by CK2 reduces DNA affinity of the FANCD2/FANCI complex, preventing binding to negatively charged DNA via electrostatic repulsion. Dephosphorylation by a phosphatase is expected to alleviate the inhibition and permit chromatin loading. Parts of this figure were made in Biorender. (B) Immunoblot analysis of HeLa cells before and after treatment with 200 nM okadaic acid (OA), collected 1, 2, or 3 h post TMP/UVA treatment. (C) Live-cell imaging of HeLa FANCD2^−/−^ cells complemented with EGFP-FANCD2 and mCherry-UHRF1 treated with 20 μg/mL TMP and irradiated by a localized laser at indicated areas (white arrows), in the presence or absence of 200 nM OA. Cells were monitored for the indicated time post-irradiation. Mean ± SEM; number of cells analyzed: 5 cells for control, 5 for OA; *n* = 3 biological replicates. Scale bar: 10 μm. (D) Combination of quantification data from (C). (E) Immunoblot analysis of HeLa EGFP-FANCD2 knockin cells before and after treatment with TMP/UVA. Cells were collected 3 h post-UVA irradiation. ^∗^*p* ≤ 0.05; ^∗∗^*p* ≤ 0.01; ^∗∗∗^*p* ≤ 0.001; ^∗∗∗∗^*p* ≤ 0.0001. See also Figure S1.

We previously found that phosphorylation of a cluster of 6 amino acids (882–898) in FANCD2 functions to inhibit loading of the FANCD2/FANCI complex onto chromosomes, resulting in a full shutdown of the FA pathway.^10^ Consequently, one could speculate that dephosphorylation of the cluster could trigger activation of the FA pathway. Supporting this theory, the cluster is indeed phosphorylated to a lesser degree following chromatin loading and monoubiquitination of the FANCD2/FANCI complex.^10^ This led us to speculate that a putative phosphatase activity might exist in humans, which can specifically dephosphorylate the cluster, licensing the FANCD2/FANCI complex for chromatin loading and thereby activating the pathway (Figure 1A). To test this hypothesis, we treated HeLa cells with a phosphatase inhibitor covering a broad family of this class of enzymes and assessed the degree of FANCD2 monoubiquitination following the introduction of ICLs. While we observed a robust monoubiquitination of FANCD2 in control cells, treatment of cells with 200 nM okadaic acid effectively blocked the increase in monoubiquitination (Figure 1B). This concentration of okadaic acid inhibits the the PP2A family of phosphatases *in vivo*,^11^ suggesting that an enzyme from this family could be responsible for dephosphorylating FANCD2.

Since monoubiquitination depends on chromatin loading, we decided to test whether okadaic acid would also affect the recruitment of FANCD2 to ICLs. We stably expressed EGFP-FANCD2, as well as mCherry-UHRF1 as a marker for ICLs, in HeLa FANCD2^−/−^ cells at levels similar to those of the endogenous proteins (Figure S1A). We then incubated cells with 4,5′,8-trimethylpsoralen (TMP) and irradiated them with a fine laser beam of UVA light at a local region in the nucleus to introduce ICLs in only that area. Thereafter, we observed the fluorescence over time using live-cell imaging. As expected, both FANCD2 and UHRF1 were strongly recruited in control cells (Figure 1C). In contrast, treating cells with okadaic acid nearly abrogated the recruitment of FANCD2, while not affecting the recruitment of UHRF1 (Figures 1C and 1D). These data show that a phosphatase activity, possibly PP2A, is important for loading FANCD2 onto chromosomes and for its monoubiquitination.

To further test whether PP2A indeed is the functional enzyme for FANCD2 dephosphorylation, we decided to genetically deplete its activity. PP2A is a trimeric holoenzyme composed of a catalytic subunit, a scaffold subunit, and a regulatory subunit. In humans, there are 2 catalytic subunits, 2 scaffold subunits, and 15 regulatory subunits. Using small interfering RNA (siRNA), we depleted the catalytic subunits, PPP2CA and PPP2CB, either individually or in combination, and assessed the ability of FANCD2 to be ubiquitinated. Strikingly, while strong monoubiquitination was observed in control cells 3 h after the introduction of ICLs (Figure 1E, lanes 1–2), FANCD2 monoubiquitination was inhibited following the depletion of PPP2CA and PPP2CB (Figure 1E, lanes 9–10). FANCD2 is ubiquitinated primarily in the S-phase of the cell cycle. Even though the duration of the experiment was relatively short, we thought it important to rule out that the cell cycle was affected by depleting PPP2CA and PPP2CB, which could bias the observed result. Fluorescence-activated cell sorting (FACS) analysis showed that changes in the proportion of S-phase cells could not account for the effect on FANCD2 monoubiquitination (Figures S1B and S1C). Taken together, using chemical inhibitors and genetics, these experiments show that PP2A activity is required for normal FANCD2 monoubiquitination.

### Recruitment of FANCD2 to chromosomes depends on PP2A

**Figure 2:**
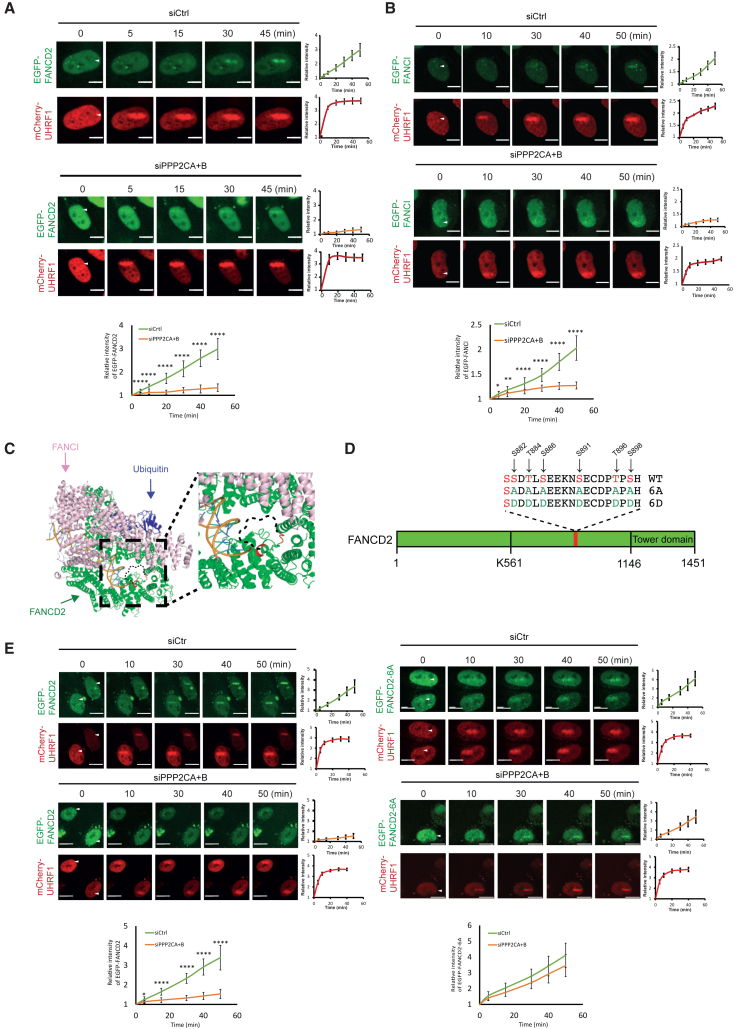
FANCD2 needs to be dephosphorylated at the CK2 cluster by PP2A prior to chromatin recruitment (A) Live-cell imaging of HeLa FANCD2^−/−^ cells complemented with EGFP-FANCD2 and mCherry-UHRF1 transfected with either a control siRNA or siRNAs targeting PP2A catalytic subunits. Mean ± SEM; number of cells analyzed: 5 cells for each group; *n* = 3 biological replicates. Scale bar: 10 μm. (B) Live-cell imaging of HeLa cells stably expressing EGFP-FANCI and mCherry-UHRF1 transfected with either a control siRNA or siRNAs targeting PP2A catalytic subunits. Mean ± SEM; number of cells analyzed: 5 cells for each group; *n* = 3 biological replicates. Scale bar: 10 μm. (C) Structure of ubiquitinated FANCD2/FANCI complex (PDB: 6VAE) bound to DNA.^12^ FANCD2 is shown in green, FANCI in pink, and ubiquitin in blue. Magnification of the area in the proximity of the 882–898 CK2 cluster. The sequence from amino acids (aa) 842 to 915, where the CK2 cluster is located, is missing from the structure; thus, this region is drawn as a black dashed line. Four amino acids right before (aa 838–841) and right after (aa 916–919) the missing area are labeled in red for visibility. (D) Schematic representation of the 882–898 six-residue cluster in FANCD2, FANCD2-6A, and FANCD2-6D. (E) Live-cell imaging of HeLa FANCD2^−/−^ cells complemented with either EGFP-FANCD2 or EGFP-FANCD2-6A and mCherry-UHRF1, followed by siRNA transfection against the PP2A catalytic subunits. Mean ± SEM; number of cells analyzed: 5 cells for each group; *n* = 3 biological replicates. Scale bar: 10 μm. ^∗^*p* ≤ 0.05; ^∗∗^*p* ≤ 0.01; ^∗∗∗^*p* ≤ 0.001; ^∗∗∗∗^*p* ≤ 0.0001. See also Figure S2.

Monoubiquitination of FANCD2 only takes place after the FANCD2/FANCI complex is loaded onto chromosomes.^3^ Our experiment using okadaic acid suggested that a PP2A activity is needed for chromatin enrichment. Since our genetic data demonstrate that PPP2CA and PPP2CB activity is required for normal monoubiquitination, we set out to test whether recruitment of FANCD2 to ICLs on chromosomes depends on PPP2CA and PPP2CB. Using siRNA, we depleted both catalytic PP2A phosphatase subunits in HeLa FANCD2^−/−^ cells stably expressing EGFP-FANCD2 and mCherry-UHRF1 (Figure S2A). Using TMP/UVA to introduce ICLs in a local region of the nucleus, we then applied live-cell imaging to monitor the recruitment of both proteins to ICLs in chromosomes over time. As expected, both proteins were strongly recruited in control cells (Figure 2A). In contrast, when we depleted PPP2CA and PPP2CB, the recruitment of FANCD2 was nearly abolished, while UHRF1, the ICL sensor protein, was recruited normally (Figure 2A).

FANCD2 functions together with FANCI as a heterodimeric complex. It has been hypothesized that FANCI activation follows and is dependent on FANCD2.^4^^,^^9^^,^^13^ If so, then we would also expect FANCI loading onto chromosomes to be affected by PP2A. To determine this directly, we established a HeLa cell line stably expressing EGFP-FANCI as well as mCherry-UHRF1 (Figure S2B). Live-cell recruitment of EGFP-FANCI has never been reported in the literature. Introducing ICLs via local TMP/UVA treatment triggered a strong recruitment of FANCI and UHRF1 (Figure 2B). As expected, depleting PPP2CA and PPP2CB diminished FANCI loading, but did not affect UHRF1 loading (Figure 2B). Thus, PP2A appears to be necessary for the chromatin loading of both FANCD2 and FANCI, presumably as a heterodimeric complex.

The cluster of 6 amino acids (882–898) in FANCD2, which we speculate is dephosphorylated by PP2A, resides in a region of 74 amino acids that has not been resolved in any of the multiple structural studies reported.^3^^,^^12^^,^^14^^,^^15^^,^^16^^,^^17^^,^^18^^,^^19^^,^^20^ We therefore suspect that the cluster lies in a flexible region, potentially a loop, which can adapt multiple conformations in close vicinity to DNA. The phosphorylated state of the cluster is then expected to repel the DNA via electrostatic repulsion. Modeling the unstructured region into the human FANCD2/FANCI complex bound to DNA shows its potential proximity to DNA (Figure 2C). To gain further insights, we then used AlphaFold2 to predict the folding of amino acids 700–1,400 of FANCD2, which harbors the phosphorylation cluster at 882–898. This resulted in 5 predictions with a very close match to the existing structure outside the 74 amino acids. In 3 of the predictions, much of the 74 amino acid loop was located close to the DNA helix, while some of the loop was located further away in 2 predictions (Figure S2C). Superimposing 1 of the 3 predictions onto the cryo-electron microscopy structure visualized how the loop containing the PP2A cluster could be located close to the DNA helix (Figure S2D). It should be noted that these are predictions, and that the actual folding and precise location of the 74 amino acids could be different. However, it is clear that the 74 amino acids are present in that region of the FANCD2/FANCI complex in some shape and that the DNA helix is also located in the same region, so it is plausible that the cluster and the DNA are within interaction distance.

PP2A might act directly on the 6 amino acids in the cluster; alternatively, the action of the phosphatase could be indirect. To test this, we mutated the 6 residues either to alanine residues, FANCD2-6A, to prevent phosphorylation, or to aspartic acid residues, FANCD2-6D, to mimic the phosphorylated state (Figure 2D). We know that the FANCD2-6D mutant has lost its DNA-binding activity, presumably via the described electrostatic repulsion of DNA by the loop harboring the 6 residues.^10^ Consequently, this mutant is not loaded onto chromatin and is not monoubiquitinated. The FANCD2-6A mutant, however, is active. If PP2A acts directly on FANCD2 by dephosphorylating the 6 residues, then one would expect the FANCD2-6A mutant to be “immune” to the depletion of PP2A, since this mutant can be neither phosphorylated nor dephosphorylated. However, if PP2A acts indirectly or via other residues in FANCD2, then the FANCD2-6A protein should also be negatively affected by PP2A depletion. We stably expressed EGFP-FANCD2-6A and mCherry-UHRF1 in HeLa cells and assessed chromatin loading of the 2 proteins to ICLs using live-cell imaging. Both EGFP-FANCD2 and EGFP-FANCD2-6A were robustly recruited in control cells (Figure 2E). Depleting PPP2CA and PPP2CB (Figure S2A) abolished the recruitment of EGFP-FANCD2, as observed in previous experiments. However, in contrast, the depletion of PPP2CA and PPP2CB had no effect on the recruitment of FANCD2-6A (Figure 2E). In good agreement, monoubiquitination of EGFP-FANCD2-6A was also unaffected by the depletion of PPP2CA and PPP2CB (Figure S2A, lanes 3–4 and 7–8) while monoubiquitination of EGFP-FANCD2 was reduced (Figure S2A, lanes 1–2 and 5–6). In other words, the EGFP-FANCD2-6A mutant is immune to the depletion of PP2A, suggesting a direct role of PP2A. Taken together, PP2A is required for chromatin loading and full monoubiquitination of the FANCD2/FANCI complex, likely via a direct dephosphorylation of FANCD2.

### PPP2R3A is the regulatory subunit of PP2A acting on FANCD2

**Figure 3:**
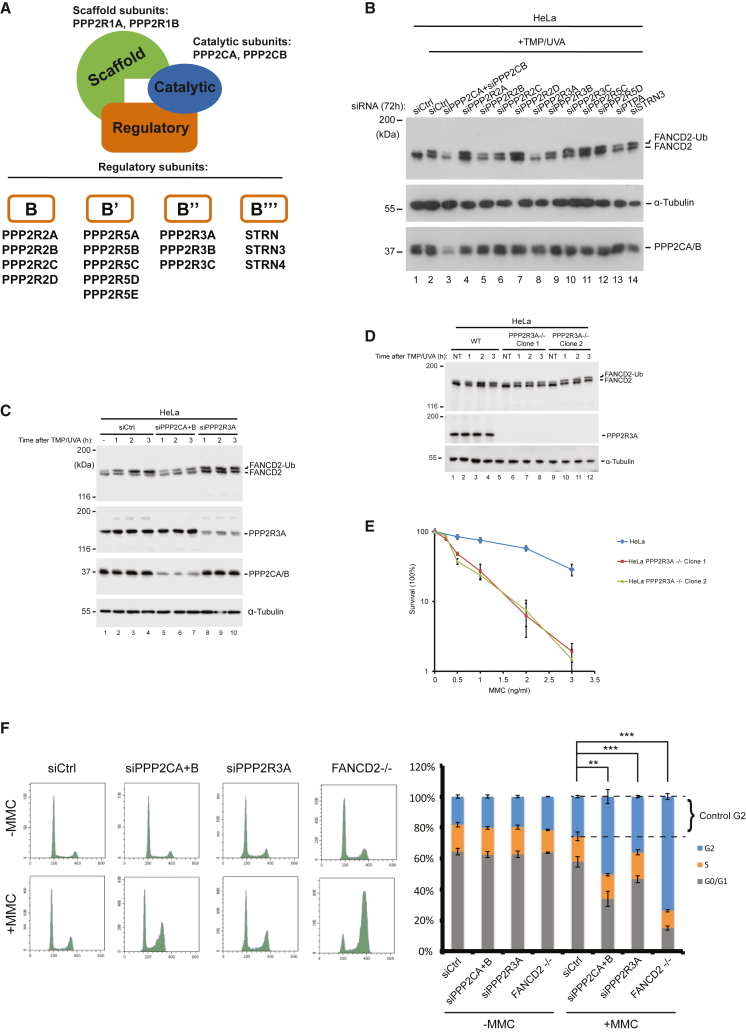
PPP2R3A/PP2A activates FANCD2 in response to ICL damage (A) The PP2A holoenzyme is a trimeric protein comprising a catalytic subunit, a scaffold subunit, and a regulatory subunit. The catalytic and scaffold subunits are encoded by only 2 genes each, while 15 genes have been identified to encode the regulatory subunit. The regulatory subunits are subdivided into 4 different families (B, B′, B″, and B‴). (B) Immunoblot analysis of HeLa cells transfected with various siRNAs targeting PP2A subunits. (C) Immunoblot analysis of HeLa cells transfected with siRNAs targeting PPP2CA/PPP2CB or PPP2R3A, followed by TMP/UVA treatment. Cells were harvested 1–3 h post-treatment. (D) Immunoblot analysis of HeLa PPP2R3A^−/−^ cells treated with TMP/UVA. Cells were harvested 1–3 h post-treatment. (E) Clonogenic survival assay of HeLa PPP2R3A^−/−^ cells treated with MMC. Colonies were counted 14 days after seeding. Mean ± SEM; *n* = 3 technical replicates. Results are confirmed with 2 independent experiments. (F) Cell-cycle analysis by FACS of HeLa cells depleted for PPP2CA/PPP2CB or PPP2R3A treated with 20 ng/mL MMC for 2 h followed by 24 h recovery. HeLa FANCD2^−/−^ cells were used as a positive control. The fractions of cells in the G2 phase were quantified. Data are means ± SDs. ^∗^*p* ≤ 0.05; ^∗∗^*p* ≤ 0.01; ^∗∗∗^*p* ≤ 0.001. See also Figure S3.

PP2A is a trimeric enzyme composed of a catalytic, a scaffold, and a regulatory subunit (Figure 3A). Specificity of the enzyme is provided by the regulatory subunit, of which there are 15 different proteins. To better understand the mechanism of how PP2A activates the FANCD2/FANCI complex, we sought to identify the regulatory subunit. To this end, we performed a targeted genetic screen against the regulatory subunits. HeLa cells were treated with individual siRNAs against the various regulatory subunits, and the FA pathway was subsequently activated by TMP/UVA treatment. Potential phenotypes displaying reduced activation of the pathway were determined by assessing the degree of FANCD2 monoubiquitination by immunoblot analysis. FANCD2 was strongly monoubiquitinated in control cells (Figure 3B, lane 2), while depletion of the catalytical subunits, PPP2CA and PPP2CB, suppressed monoubiquitination (Figure 3B, lane 3), in good agreement with our previous experiments. Of the various depleted regulatory subunits, the protein that gave the strongest phenotype was PPP2R3A (Figure 3B, lane 8). In fact, FANCD2 monoubiquitination was very similarly reduced when depleting either PPP2CA/PPP2CB or PPP2R3A (Figure 3B, lanes 3 and 8). To further examine the putative functional role of PPP2R3A as the active subunit of PP2A, we performed a time course, assessing FANCD2 monoubiquitination over time in the presence or absence of PPP2R3A after the introduction of ICLs by TMP/UVA treatment. In control cells we observed a very strong monoubiquitination, with the vast majority of FANCD2 being monoubiquitinated at the 2-h time point (Figure 3C, lane 3). In contrast, depleting either PPP2CA/PPP2CB or PPP2R3A resulted in only a minority of FANCD2 being monoubiquitinated at the 2-h time point (Figure 3C, lanes 6 and 9). To further test these findings, we depleted PPP2R3A using a different method, CRISPR-Cas9-mediated knockout (Figure S3A). Again, we observed a pronounced reduction in FANCD2 monoubiquitination in 2 independent HeLa PPP2R3A −/− clones (Figure 3D).

The clear phenotype of reduced FANCD2 monoubiquitination suggests that the FA pathway could be similarly affected, which would lead to a weakened cellular response to ICLs. If so, we would expect PPP2R3A −/− cells to be sensitized to ICL-forming drugs, such as mitomycin C (MMC). Thus, we tested the sensitivity of the 2 independent knockout cell lines to MMC using a clonogenic survival assay. Both cell lines were hypersensitive toward MMC when compared to control cells (Figure 3E).

Human cells with an inactive FA pathway accumulate in the G2-phase of the cell cycle following exposure to low concentrations of MMC, due to the failure to repair ICLs and consequent incomplete replication preventing chromosome segregation. To test whether depleting PP2A also causes such an FA-like phenotype, we depleted either the catalytic or the regulatory subunits of PP2A by siRNA (Figure S3B) and assessed the cell-cycle profiles by FACS analysis. Depleting either PPP2CA/PPP2CB or PPP2R3A caused a significant increase in G2-arrested cells compared to control cells (Figure 3F).

There are 2 PP2A scaffold proteins in humans, PPP2R1A and PPP2R1B. We assessed the contribution of these 2 subunits toward the PP2A activity acting on FANCD2 in live cells. To this end, we reduced the cellular concentrations of either factor individually, or in combination, and evaluated the effect on FANCD2 monoubiquitiation. We observed an attenuation of ubiquitination resulting from reducing either of the scaffold factors, suggesting some contribution from both of them (Figure S3C).

Taken together, these data show that PPP2R3A, as the regulatory subunit of PP2A, is important for the activation of FANCD2 in live cells.

### PPP2R3A is required for FANCD2 loading onto chromosomes

**Figure 4:**
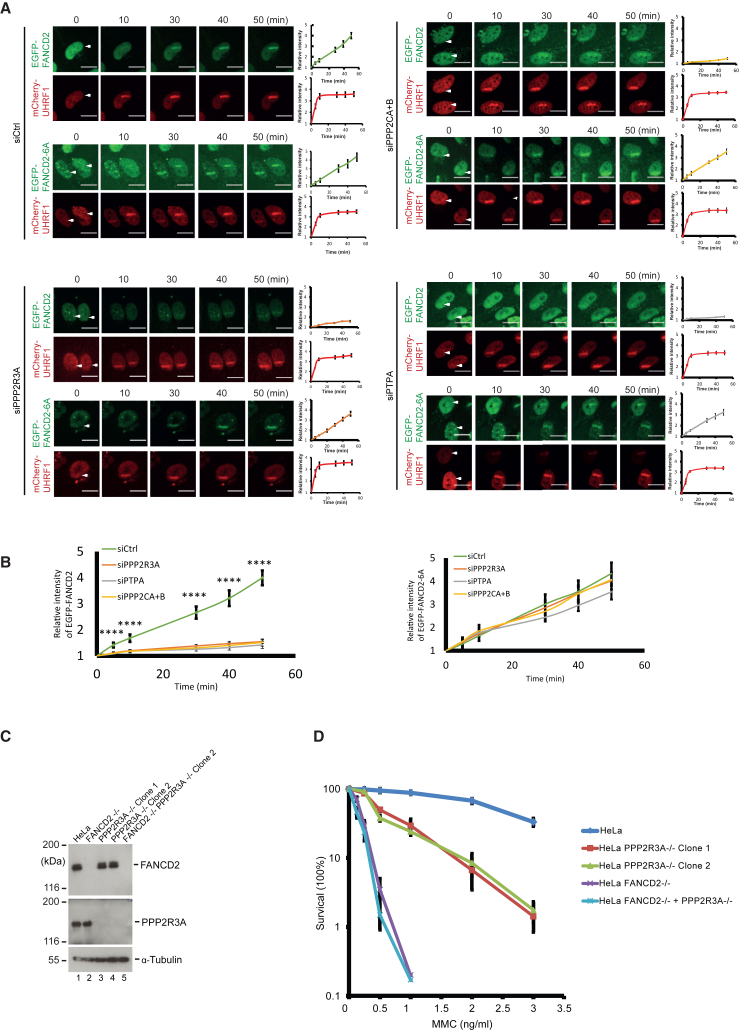
PPP2R3A/PP2A is functionally important for the activation of the FA pathway (A) Live-cell imaging of HeLa FANCD2^−/−^ cells complemented with either EGFP-FANCD2 or EGFP-FANCD2-6A and mCherry-UHRF1 transfected with either a control siRNA or siRNAs targeting PPP2CA/PPP2CB, PPP2R3A, or PTPA. Mean ± SEM; number of cells analyzed: 5 cells for each group; *n* = 3 biological replicates. Scale bar: 10 μm. (B) Combination of quantification data from (A). (C) Immunoblot analysis of HeLa cells, where either *FANCD2*, *PPP2R3A*, or both *FANCD2* and *PPP2R3A* genes were disrupted using CRISPR-Cas9. (D) Clonogenic survival assay of HeLa, HeLa FANCD2^−/−^, HeLa PPP2R3A^−/−^, and HeLa FANCD2^−/−^ + PPP2R3A^−/−^ clones treated with indicated concentrations of MMC. Mean ± SEM; *n* = 3 technical replicates. ^∗^*p* ≤ 0.05; ^∗∗^*p* ≤ 0.01; ^∗∗∗^*p* ≤ 0.001; ^∗∗∗∗^*p* ≤ 0.0001.

Our data show that PPP2R3A is necessary for the normal monoubiquitination of FANCD2. We speculated that PPP2R3A might therefore also control the loading of FANCD2 onto chromosomes, since monoubiquitination requires prior binding of the FANCD2/FANCI complex to DNA. Using specific introduction of ICLs in local regions of individual cells via TMP/UVA, we could observe the recruitment of the FANCD2/FANCI complex to ICLs on chromosomes by live-cell imaging. Using this system, we next assessed the effect of depleting PPP2R3A. As predicted, depletion of PPP2R3A caused a pronounced decrease in the recruitment of EGFP-FANCD2, while mCherry-UHRF1 was unaffected (Figures 4A and 4B). EGFP-FANCD2 and mCherry-UHRF1 were recruited normally in control cells in these experiments (Figure 4A). The observed suppression mirrored the effect seen when depleting the PP2A catalytic subunits PPP2CA/PPP2CB (Figures 4A and 4B). As an additional control, we depleted PTPA, which is a protein required for the biogenesis of PP2A.^21^ Reducing the cellular level of PTPA is expected to cause a reduction in cellular PP2A activity. When we depleted PTPA, again, we observed a near abrogation of EGFP-FANCD2 recruitment, phenocopying the depletion of PPP2R3A. Importantly, recruitment of the EGFP-FANCD2-6A mutant is unaffected by depletion of both PPP2R3A and PTPA (Figure 4A). These data reinforce the notion that PP2A activity, more specifically the PPP2CA/PPP2R1A/PPP2R3A complex, is important for FANCD2 loading onto chromosomes and monoubiquitination (Figures 4A and 4B).

Our data demonstrate a correlation between PP2A activity and the activation of FANCD2 in live cells. However, PP2A might also act on other proteins within the FA pathway. If FANCD2 is the primary protein in the FA pathway that PP2A regulates, then one could speculate that if cells do not possess FANCD2, then depletion of PP2A should not sensitize such cells further toward MMC. To test this hypothesis, we depleted either FANCD2 or PPP2R3A separately or in combination by CRISPR-Cas9 knockout. Both genes were efficiently disrupted in the resulting cell lines (Figure 4C). We then subjected the established knockout cell lines to a clonogenic survival assay using MMC to introduce ICLs. We observed that FANCD2-deficient cells were sensitive to MMC and that additional depletion of PPP2R3A did not further sensitize the cells (Figure 4D). As depletion of FANCD2 abrogates the FA pathway, we can therefore conclude that PP2A functions within the FA pathway, possibly acting directly on FANCD2.

Taken together, these data show that PP2A, with PPP2R3A as a regulatory subunit, is critical for FANCD2 chromosome loading and for its activity in ICL repair.

### PP2A (PPP2CA/PPP2R1A/PPP2R3A complex) dephosphorylates FANCD2 directly

**Figure 5:**
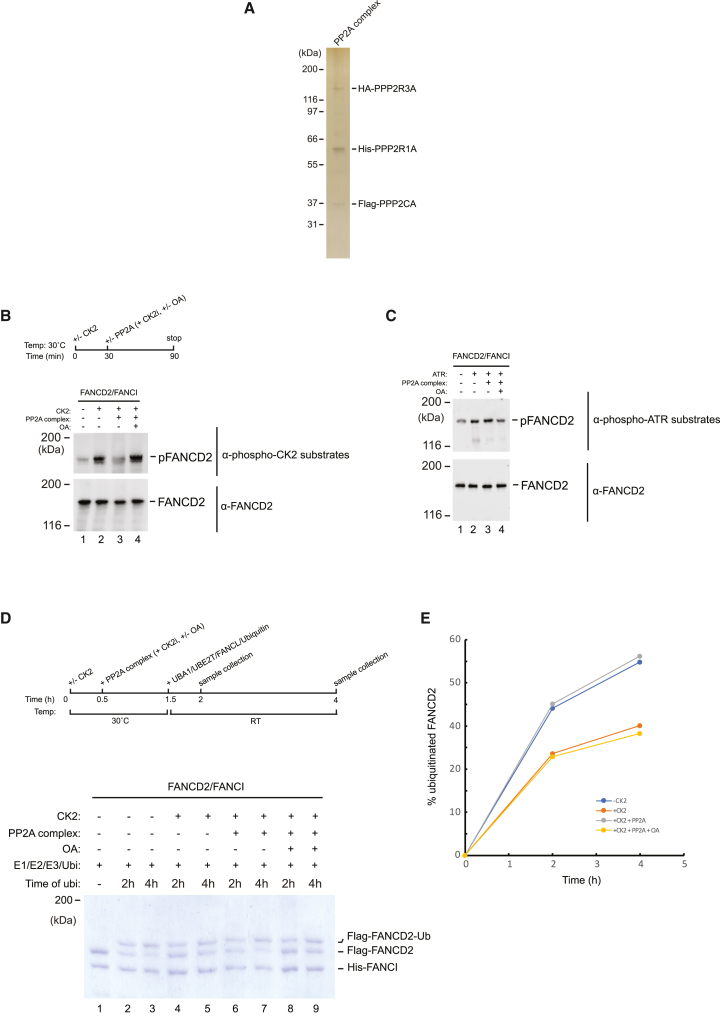
Recombinant PP2A (PPP2CA/PPP2R1A/PPP2R3A complex) dephosphorylates FANCD2 and promotes its monoubiquitination and DNA binding *in vitro* (A) Silver stain of recombinant PPP2CA/PPP2R1A/PPP2R3A holoenzyme complex purified from Sf9 insect cells. (B) Immunoblot analysis of the FANCD2/FANCI complex subjected to an *in vitro* phosphorylation/dephosphorylation assay. The FANCD2/FANCI complex was first phosphorylated by CK2, followed by dephosphorylation by the PPP2CA/PPP2R1A/PPP2R3A enzyme complex. (C) Immunoblot analysis blot of a phosphorylation/dephosphorylation similar to what is shown in (B), except that recombinant ATR was used instead of CK2. (D) *In vitro* ubiquitination assay of the FANCD2/FANCI complex phosphorylated by CK2, followed by dephosphorylation by the PPP2CA/PPP2R1A/PPP2R3A enzyme complex as indicated. A schematic diagram of the experimental procedure is shown. Coomassie brilliant blue stain. A representative of 3 independent experiments is shown. (E) Quantification of data presented in (D) showing the percentage of FANCD2-Ub. See also Figure S4.

Our data thus far show that PP2A activity is important for loading FANCD2 onto chromosomes in live cells and for its subsequent activatory monoubiquitination. Although the various types of experiments suggest that PP2A itself directly dephosphorylates FANCD2 to enable these processes, as opposed to its activity being required via the dephosphorylation of intermediate factors, none of the data show this unequivocally. Therefore, we decided to recapitulate the dephosphorylation reaction *in vitro*, using purified components. We cloned the catalytic subunit PPP2CA, the scaffold subunit PPP2R1A, and the regulatory subunit PPP2R3A in expression vectors for expression in Sf9 insect cells. By a combination of affinity chromatography, gel filtration, and ion exchange, we succeeded in purifying a pure trimer of PPP2CA/PPP2R1A/PPP2R3A, the full PP2A holoenzyme complex (Figure 5A). To test whether the PP2A complex could specifically dephosphorylate FANCD2, we first purified the FANCD2/FANCI complex to homogeneity from Sf9 cells (Figure S4A). We phosphorylated this FANCD2/FANCI complex using recombinant CK2. In this reaction, we observed a strong phosphorylation of FANCD2 (Figure 5B, lane 2). We then subjected the phosphorylated FANCD2 to a dephosphorylation reaction using the purified PP2A complex (PPP2CA/PPP2R1A/PPP2R3A). As expected, we observed a clear dephosphorylation of FANCD2 (Figure 5B, lane 3). The addition of okadaic acid could block the reaction, demonstrating that the dephosphorylation was specific (Figure 5B, lane 4). To further test the specificity of our purified PP2A complex, we phosphorylated the FANCD2/FANCI complex by ATR and assessed whether our PP2A phosphatase could also reverse this phosphorylation. We observed no dephosphorylation of the ATR sites by the purified PP2A complex, demonstrating the specificity of the phosphatase complex (Figure 5C).

Our experiments using live cells show that PP2A is important for FANCD2 loading onto chromosomes and its subsequent monoubiquitination, while our reconstituted reactions *in vitro* show that PP2A directly dephosphorylates FANCD2. Our interpretation of these data is that PP2A dephosphorylates FANCD2 on its CK2 cluster, thereby relieving it from the inhibitory phosphorylation of this cluster, which electrostatically repulses the DNA helix. If our hypothesis is correct, then CK2 phosphorylation of FANCD2 should reduce its propensity to be monoubiquitinated, while a subsequent PP2A dephosphorylation should restore its ability to be monoubiquitinated. To test this directly, we set out to reconstitute the full and coupled phosphorylation, dephosphorylation, and monoubiquitination reactions *in vitro* using purified proteins. We purified the E1 ubiquitin-activating enzyme (UBA1), the E2 ubiquitin-conjugating enzyme (UBE2T), and the E3 ubiquitin ligase (FANCL) to homogeneity (Figure S4B). We then subjected the FANCD2/FANCI complex to an *in vitro* monoubiquitination reaction, observing a clear monoubiquitination (Figures 5D, lanes 2–3, and 5E). We then phosphorylated the FANCD2/FANCI complex with CK2 prior to the monoubiquitination reaction, and as expected, we observed a reduction in monoubiquitination (Figures 5D, lanes 4–5, and 5E). We then incubated the FANCD2/FANCI complex with our PP2A complex following CK2 phosphorylation and observed a complete restoration of monoubiquitination (Figures 5D, lanes 6–7, and 5E), demonstrating that PP2A could reverse the inhibition mediated by CK2 phosphorylation. The restoration was completely prevented by the inclusion of okadaic acid, the PP2A inhibitor, in the reaction (Figures 5D, lanes 8–9, and 5E).

These data show that the PP2A complex, composed of PPP2CA/PPP2R1A/PPP2R3A, could specifically dephosphorylate FANCD2 that was previously phosphorylated by CK2, and that this dephosphorylation activated the complex, permitting it to be monoubiquitinated.

### Super-resolution single-molecule tracking in live cells shows that PP2A licenses genome-wide chromosome loading of FANCD2

**Figure 6:**
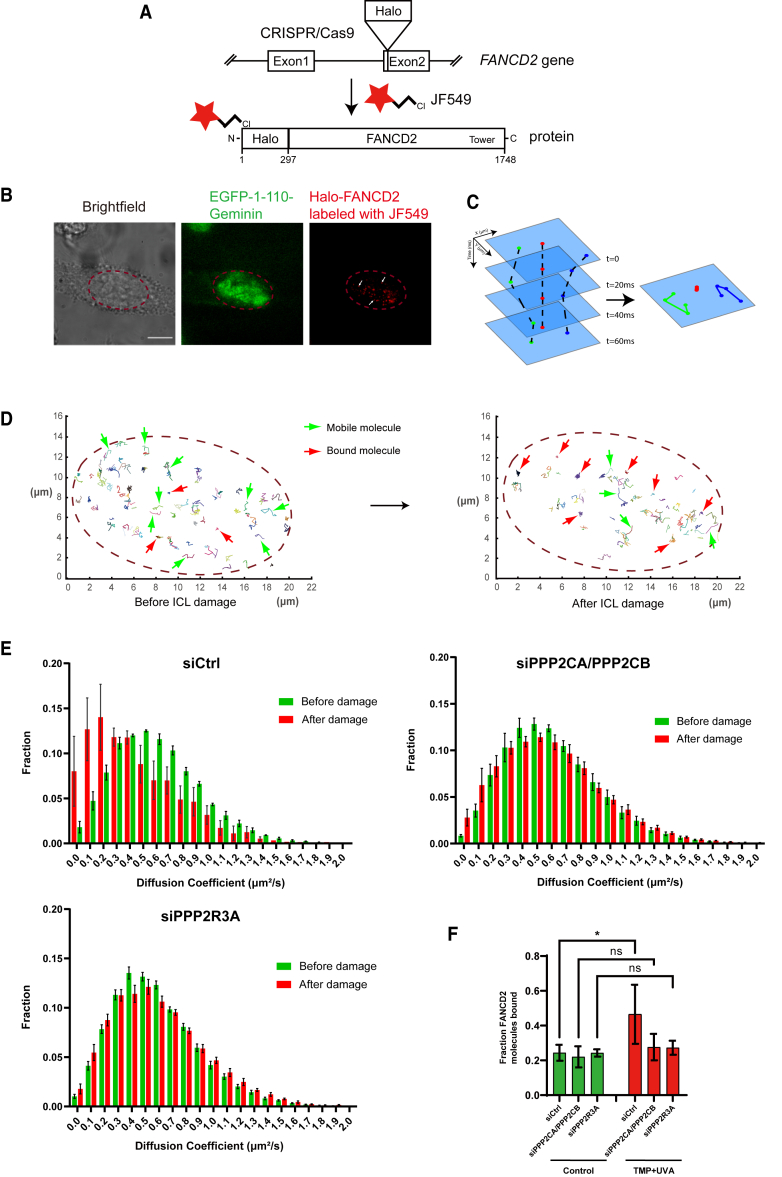
Super-resolution single-molecule tracking shows that PP2A controls genome-wide chromosome loading of FANCD2 (A) Schematic representation showing the generation of a Halo-FANCD2 knockin HeLa cell line using CRISPR-Cas9. The HaloTag was integrated into the reading frame at the ATG start codon located in exon 2 of the *FANCD2* gene via gene editing. Full HaloTag-FANCD2 knockin cells were then stably transfected with EGFP-1-110-Geminin. (B) Super-resolution microscopy images show nuclear EGFP-1-110-Geminin and Halo-FANCD2 molecules. S-/G2-phase cells with strong EGFP signals were selected and imaged. Scale bar: 10 μm. (C) Schematic showing how the movement of individual FANCD2 molecules can be imaged and tracked over time. The 2 molecules indicated in green or blue are mobile, while the molecule indicated in red is bound. For clarity, only 4 frames are shown. A recording typically consists of 4,000 frames. (D) A subset of trajectories of FANCD2 molecules upon unperturbed and TMP/UVA-treated conditions. The trajectories of molecules present for at least 5 frames (100 ms), generated from 50 frames from a 4,000-frame video. (E) Distribution of diffusion coefficients of FANCD2 molecules that possess at least 5 localizations per track. The data are split into 2 populations based on the diffusion coefficient per track, bound FANCD2 molecules (D ≤ 0.3 μm^2^/s), and mobile FANCD2 molecules (D > 0.3 μm^2^/s) (3 cells per treatment, tracks >5,000 per cell). Data are means ± SDs. Cells are transfected with siRNA 72 h before imaging. *n* = 3 biological replicates. (F) Fraction of bound FANCD2 molecules in each treatment in (E). Data are means ± SDs. ^∗^*p* ≤ 0.05 (ANOVA). See also Figure S5.

Our data show that PP2A activates the FANCD2/FANCI complex by enabling it to load onto ICLs on chromosomes, which permits subsequent monoubiquitination. We have shown this using a range of complementary experimentation both *in vitro* and *in vivo*. Common to all these experiments is that we measure an average of many molecules. Thus, we decided to establish an experimental system allowing us to observe and measure individual FANCD2 molecules in live cells. To this end, we set up super-resolution live-cell single-particle tracking. We used CRISPR-Cas9 to insert a Halo tag into the *FANCD2* gene in frame with the N terminus of the FANCD2 protein (Figure 6A). The Halo tag permits labeling and visualization of individual FANCD2 molecules in live cells using strong chemical fluorophores such as JF549. The FA pathway is primarily active in the S-/G2-phase of the cell cycle. Thus, to be able to accurately assess and quantify the behavior of FANCD2 in cells specifically in the S-/G2-phase, we decided to stably express a cell-cycle marker in the same cells that already express Halo-FANCD2. A fusion protein between EGFP and the 110 N-terminal amino acids of Geminin is present in S-/G2-phase cells, but absent from G1 cells, permitting a clear determination of the cell-cycle stage of individual cells monitored in the microscope.^22^ We then observed the established cell line using a PALM/TIRF super-resolution microscope. We could easily identify S/G2 cells, which displayed a bright EGFP signal. We could also specifically observe individual FANCD2 molecules in the cells labeled with the JF549 red fluorophore (Figure 6B). As expected, both EGFP-1-110-Geminin and Halo-FANCD2 were localized in the nucleus.

By imaging cells every 20 ms, we can then track the movement of individual FANCD2 molecules over time, allowing us to plot their tracks of movement (Figure 6C). We used this approach and plotted the tracks of individual FANCD2 molecules in different colors to facilitate visualization of the trajectories of individual proteins (Figure 6D). Here, it is visible that some molecules move freely, indicated by green arrows, while other molecules are immobile, indicated by red arrows. We then introduced ICLs in cells and assessed whether the mobility profile had changed. It became apparent that the number of mobile molecules was reduced, while the number of immobile molecules had increased (Figure 6D).

The population of FANCD2 that is bound to chromosomes genome-wide can then be quantified by tracking thousands of molecules. By calculating the displacement of each molecule over time, we can then calculate the apparent diffusion coefficient (D, μm^2^/s). Molecules bound to chromosomes will have diffusion coefficients from 0 to 0.3 μm^2^/s, while mobile molecules will have diffusion coefficients greater than 0.3 μm^2^/s.^9^

Using this approach, we observed a distribution of diffusion coefficients for FANCD2 molecules in HeLa cells, with a peak of 0.5 μm^2^/s (Figure 6E, green bars; Video S1). After introducing ICLs using TMP/UVA, we observed a clear shift of diffusion coefficients, reflecting the increase in population of molecules bound to chromosomes (Figure 6E, red bars; Video S2).

Video S1. Time-lapse of a HeLa cell treated with siCtrl shown in Figure 6E Time between each frame is 20 ms. The total time of recording is 10 s. Scale bar = 5 μm.

Video S2. Time-lapse of a HeLa cell treated with siCtrl, followed by treatment with TMP/UVA shown in Figure 6E Time between each frame is 20 ms. The total time of recording is 10 s. Scale bar = 5 μm.

Using this imaging system, we could now test whether the genome-wide chromosome loading of FANCD2 following the introduction of ICLs depends on PP2A activity. We depleted either the catalytic subunits PPP2CA/PPP2CB or the specific regulatory subunit PPP2R3A in the HeLa cells expressing Halo-FANCD2 and EGFP-1-110-Geminin (Figure S5A) and subjected the resulting cells to the described super-resolution imaging and single-particle tracking. Depleting PPP2CA/PPP2CB completely prevented the increase in genome-wide chromosome loading of FANCD2 following the introduction of ICLs (Figures 6E and 6F; Videos S3 [control] and S4 [siRNA]). Likewise, depleting PPP2R3A also prevented the chromosome loading of FANCD2 following the introduction of ICLs (Figures 6E and 6F; Videos S5 [control] and S6 [siRNA]).

Video S3. Time-lapse of a HeLa cell treated with siPPP2CA/PPP2CB, shown in Figure 6E Time between each frame is 20 ms. The total time of recording is 10 s. Scale bar = 5 μm.

Video S4. Time-lapse of a HeLa cell treated with siPPP2CA/PPP2CB, followed by treatment with TMP/UVA, shown in Figure 6E Time between each frame is 20 ms. The total time of recording is 10 s. Scale bar = 5 μm.

Video S5. Time-lapse of a HeLa cell treated with siPPP2R3A, shown in Figure 6E Time between each frame is 20 ms. The total time of recording is 20 s. Scale bar = 5 μm.

Video S6. Time-lapse of a HeLa cell treated with siPPP2R3A, followed by treatment with TMP/UVA, shown in Figure 6E Time between each frame is 20 ms. The total time of recording is 10 s. Scale bar = 5 μm.

Taken together, these data show that PP2A is critical for the genome-wide chromatin loading of FANCD2 in response to ICLs introduced into the genome.

## Discussion

**Figure 7:**
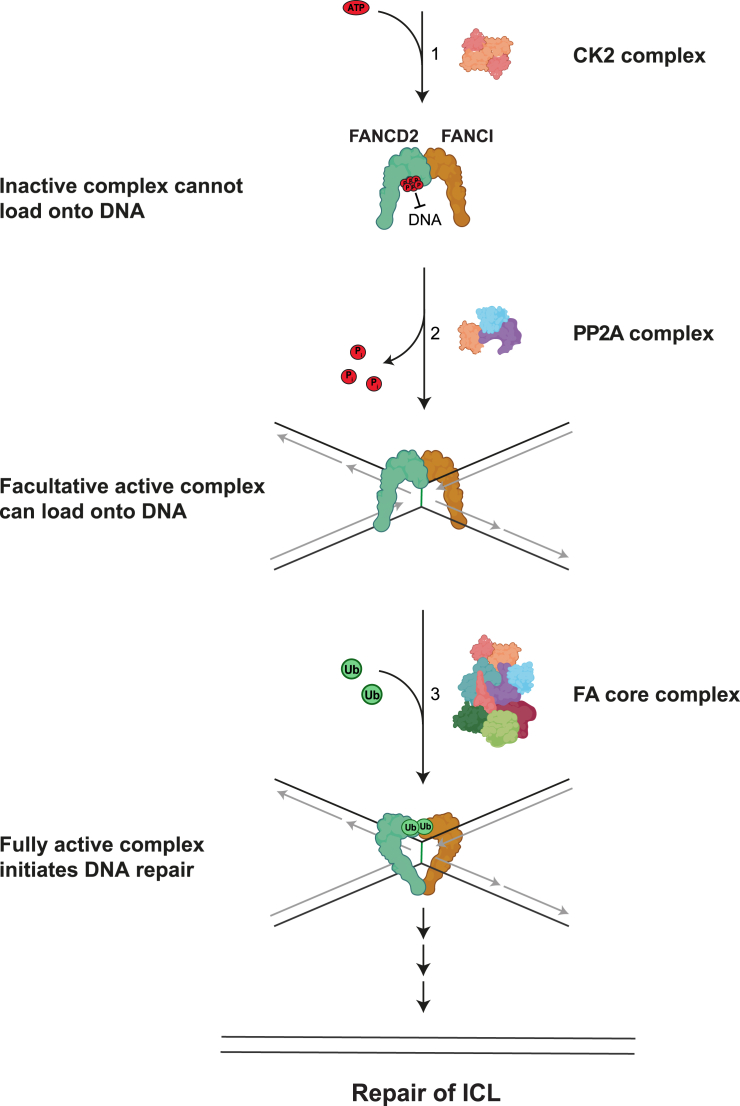
Model of how PP2A activates the FA pathway (1) In the absence of ICL damage, FANCD2 is phosphorylated by CK2, hence keeping the FANCD2/FANCI complex in a state with low DNA affinity and away from DNA via electrostatic repulsion. (2) When ICLs are induced, PP2A dephosphorylates FANCD2 at the CK2 sites, thereby switching the FANCD2/FANCI complex to a state of higher DNA affinity, which allows the complex to be loaded onto chromatin. (3) The FANCD2/FANCI complex undergoes further modification after chromatin loading, including monoubiquitination by the FA core complex, which converts the complex into a sliding clamp locked on DNA. This fully activates the FA pathway by promoting the recruitment of downstream repair factors, which results in repair of the ICL damage. Parts of this figure contain elements from Biorender.

Our study reports the discovery of the PP2A phosphatase as a critical regulator of the FA pathway. Mechanistically, PP2A relieves the inhibitory phosphorylation of a cluster in FANCD2 residing in 882–898, thereby licensing chromatin loading of the FANCD2/FANCI complex, which triggers the activation of the FA pathway (Figure 7).

### Molecular switch mediated by phosphorylation and dephosphorylation of FANCD2

Our previous work uncovered how the phosphorylation of 6 residues within the 882–898 cluster of FANCD2 efficiently prevented loading the FANCD2/FANCI complex onto chromosomes by electrostatic repulsion of DNA.^10^ A mutant of FANCD2, where the cluster could not be phosphorylated (the 6 phosphorylation sites in the cluster were mutated to alanine residues), was constitutively active and resulted in uncontrolled loading of the FANCD2/FANCI complex onto chromosomes. In contrast, a mutant of FANCD2 where the cluster mimicked the phosphorylated state (the 6 phosphorylation sites in the cluster were mutated to aspartic acid residues) was inactive, and the FANCD2/FANCI complex could not be loaded onto chromosomes. The wild-type non-ubiquitinated form of FANCD2 that has not been loaded onto chromosomes was phosphorylated to a greater degree than the active monoubiquitinated form that is loaded.

These findings clearly demonstrate how phosphorylation of the cluster controls the loading of the FANCD2/FANCI complex onto chromosomes. However, it is also clear that for the complex to be both switched on and off via the phosphorylation status of this cluster, both a kinase and a phosphatase would be required. The present discovery of PP2A resolves this conundrum.

### PP2A specifically dephosphorylates FANCD2 and activates the FANCD2/FANCI complex *in vivo* and *in vitro*

We found that loading of the FANCD2/FANCI complex onto chromosomes was dependent on the enzymatic activity of PP2A. Either inhibiting PP2A using small-molecule inhibitors or by depleting PP2A either using RNAi or full knockout of the regulatory subunit, PPP2R3A, suppressed recruitment of the FANCD2/FANCI complex to ICLs on chromosomes. Cells with reduced PP2A activity are sensitive to ICL-forming drugs. Importantly, a mutant of FANCD2, where the phosphorylation cluster is mutated to alanines and therefore can be neither phosphorylated nor dephosphorylated, is immune to any interference of cellular PP2A activity, demonstrating that PP2A acts directly on the FANCD2/FANCI complex.

Loading of the FANCD2/FANCI complex onto chromosomes is followed by the important monoubiquitination, which results in a conformational change of the complex to a more closed form, encircling the DNA.^12^^,^^14^^,^^17^ Thus, the ubiquitination reaction is dependent on prior DNA loading for it to take place. We were able to recapitulate these reactions *in vitro*, using purified components. We observed that phosphorylating the cluster reduced the degree of monoubiquitination. Importantly, if we incubated the phosphorylated FANCD2/FANCI complex with our highly purified PP2A trimer enzyme complex (PPP2CA/PPP2R1A/PPP2R3A), then the inhibition was completely reversed. This reinforces the notion that PP2A is the enzyme activating the FANCD2/FANCI complex following its prior inhibition by phosphorylation of the cluster.

### ATR phosphorylation and PP2A dephosphorylation

We recently discovered a number of new ATR phosphorylation sites in FANCD2.^9^ As opposed to the inhibitory effect of phosphorylating the 882–898 cluster, phosphorylation of the ATR sites stimulates the activation of the FANCD2/FANCI complex, facilitating its loading onto chromosomes. It has also been reported that FANCI is phosphorylated by ATR in a stimulatory way.^6^^,^^8^ Some of the ATR sites on FANCD2 are located on the surface of the complex, while the exact position of others are unresolved in published structures. Phosphorylation of the ATR sites enhances the DNA binding of the FANCD2/FANCI complex, although it is likely that there are additional mechanisms contributing to the positive effect of these phosphorylation events in live cells. More work is needed to uncover how the various newly identified sites contribute to the activation of the FANCD2/FANCI complex.

What is clear, however, is that phosphorylation of some sites by ATR and dephosphorylation of other sites by PP2A are both required for loading of the FANCD2/FANCI complex onto chromosomes and its activation. The order of these events, dephosphorylation by PP2A and phosphorylation by ATR, however, is not clear, as they could be sequential or could take place in parallel. More research is required to resolve this question.

If the 2 categories of events are sequential, then it could be that one affects the other. In other words, it is possible that phosphorylation by ATR can only take place after dephosphorylation by PP2A or vice versa. Clarifying this question will be important to fully understand how the molecular switch operates in live cells.

### Super-resolution microscopy of FANCD2 in S-phase cells

We show how PP2A is required for genome-wide loading of the FANCD2/FANCI complex to ICLs. To show this, we used super-resolution single-molecule imaging in live cells. Following our recent work on how ATR phosphorylates the FANCD2/FANCI complex,^9^ this is the second example in the literature where this approach is used to study FA proteins or ICL repair in general. We observed that upon introducing ICLs into chromosomes, individual FANCD2 molecules loaded onto chromosomes, shown by their transition from a mobile to an immobile state. This transition, or loading, was dependent on PP2A. Using this approach, we can now further interrogate the fine mechanism of FANCD2/FANCI activation in live cells, with a resolution far above what we have hitherto been able to achieve.

Most likely, the FA pathway is only active during the S-phase; therefore, we analyzed only S-phase cells in these super-resolution studies. At present, we do not know exactly how PP2A is activated or recruited to the FANCD2/FANCI complex. It is possible that these regulatory events are linked to the cell cycle; however, more work will be needed to clarify this.

### Potential broader implications

FANCD2 functions in other biological processes in addition to ICL repair. For instance, it is recruited to fragile sites where it facilitates their replication,^23^ and it participates in resolving R-loops resulting from transcription-replication conflicts.^24^^,^^25^ Common to these activities is the interaction between FANCD2 and nucleic acids. We show how PP2A is critical for licensing the loading of the FANC2/FANCI complex onto ICLs on chromosomes, but it is still unknown whether other biological processes also involving the interaction of this complex with nucleic acids are also controlled by PP2A. There could exist other specific phosphatases that act to license the FANCD2/FANCI complex for loading onto other specific nucleic acid structures.

It will be interesting to see whether other DNA repair proteins that are also loaded onto chromosomes are also regulated by similar mechanisms, either by PP2A utilizing other regulatory subunits or perhaps entirely different phosphatases.

### Clinical implications

FA patients have a very high cancer predisposition. The risk of cancer can be up to several thousand-fold higher than in the general population, depending on the type of cancer and the FA gene mutated in the patient.^26^ Perhaps not surprising, the FA pathway is sometimes inactivated in sporadic cancer, believed to contribute to tumorigenesis. Furthermore, because of the toxicity of ICL-forming agents, they are used successfully in the non-FA group of cancer patients. However, often, cancers become resistant after initial responsiveness. If the FA pathway could be targeted in these resistant cancers, then it might provide therapeutic opportunities. Targeting the FA pathway could therefore be clinically relevant, either as monotherapy or combination therapy, as has been the case for poly (ADP-ribose) polymerase inhibitors.^27^^,^^28^ Since our work defines the PP2A phosphatase as critical for the activation of the FA pathway, targeting this enzyme could therefore open therapeutic opportunities.

### Conclusion

Here, we identify the PP2A phosphatase as a critical enzyme licensing the FANCD2/FANCI complex for chromatin loading. PP2A acts directly on FANCD2, relieving it from inhibitory phosphorylation events in a cluster residing in amino acids 882–898, and thereby switches on the FA pathway. Suppressing PP2A activity hinders loading of the FANCD2/FANCI complex, reduces its monoubiquitination, and deregulates the FA pathway.

### Limitations of the study

While we show that the phosphorylation status of the 882–898 cluster of FANCD2 is central to the regulation of the FANCD2/FANCI complex, we do not know how many, or which, of the 6 residues in the cluster are phosphorylated in 1 molecule of FANCD2 at one time in living cells.

## Resource availability

### Lead contact

Further information and requests for resources and reagents should be directed to and will be fulfilled by the lead contact, Martin A. Cohn (martin.cohn@bioch.ox.ac.uk).

### Materials availability

Materials will be provided upon request to the lead contact.

### Data and code availability

• The data reported in the paper will be shared upon request to the lead contact.
• This study did not generate original code.
• Any additional information required to reanalyze the data reported in this paper is available from the lead contact upon request.

## Acknowledgments

The authors would like to thank members of the Cohn laboratory for reading and discussing the manuscript. We are grateful to Stephan Uphoff for valuable advice on single-particle tracking. We are also grateful to staff at the Micron Bioimaging Facility for excellent help and support. We thank Luke Lavis (Janelia Research Campus) for providing the JF549 Halo ligand dye. This work was supported by grants UF100717 and UF150651 from the 10.13039/501100000288Royal Society (to M.A.C.), grants 153/092 and 0012197 from John Fell Oxford University Press Research Fund (to M.A.C.), grants OCRC 0213-MC and CRUKDF 0715-MC from the OCRC/CR-UK (to M.A.C.), a grant from the 10.13039/100012335Medical Research Fund (to M.A.C.), grant MR/N021002/1 from the 10.13039/501100000265Medical Research Council (to M.A.C.), a Wellcome Trust Senior Research Fellowship, 210640/Z/18/Z (to M.A.C.), an MRC-Clarendon Scholarship (to D.L.M.), a “Mutua Madrileña” Graduate Scholarship (to D.L.M.), a fellowship from the 10.13039/100008732Uehara Memorial Foundation (to A.J.-M.), and a Leathersellers’ Company Scholarship via St. Catherine’s College Oxford (to L.J.C.).

## Author contributions

D.Y., F.B., D.L.M., H.X., A.J.-M., L.J.C., and M.A.C. designed the experiments. D.Y., F.B., D.L.M., A.J.-M., L.J.C., and H.X. performed the experiments and analyzed the data. D.Y., F.B., D.L.M., H.X., A.J.-M., L.J.C., and M.A.C. prepared the manuscript.

## Declaration of interests

The authors declare no competing interests.

## STAR★Methods

### Key resources table

**Table:** 

REAGENT or RESOURCE	SOURCE	IDENTIFIER
**Antibodies**		

Mouse monoclonal anti-FANCD2 (FI17)	Santa Cruz Biotechnology	Cat# sc-20022; RRID: AB2278211
Rabbit polyclonal anti-FANCI	FARF	N/A
Mouse monoclonal anti-α-Tubulin (DM1A)	Sigma-Aldrich	Cat# 05–829; RRID: AB310035
Mouse monoclonal anti-UHRF1 (H-8)	Santa Cruz Biotechnology	Cat# sc-373750; RRID: AB10947236
Mouse monoclonal anti-PPP2CA	BD Biosciences	Cat# 610555; RRID: AB397909)
Rabbit polyclonal anti-PPP2R3A	Sigma-Aldrich	Cat# HPA035829 RRID: AB10696513)
Rabbit multi-monoclonal anti-phospho-CK2 substrate motif [(pS/pT)DXE]	Cell Signaling	Cat# 8738; RRID: AB2797653
Mouse monoclonal anti-PP2A-Aα/β (PPP2R1A/PPP2R1B) (4G7)	Santa Cruz Biotechnology	Cat# sc13600; RRID: AB628178
Rabbit multi-monoclonal anti-phospho-ATM/ATR substrate motif [(pS/pT)QG]	Cell Signaling	Cat# 6966; RRID: AB10949894
Rabbit anti-mouse immunoglobulins (horseradish peroxidase conjugated)	Dako-Agilent	Cat# P0260; RRID: AB2636929
Goat anti-rabbit immunoglobulins (horseradish peroxidase conjugated)	Dako-Agilent	Cat# P0448; RRID: AB2617138

**Bacterial and virus strains**		

Top10	Thermo Fisher	Cat# 404010

**Chemicals, peptides, and recombinant proteins**		

FuGENE6	Promega	Cat# E2691
Lipofectamine RNAiMAX	Thermo Fisher	Cat# 12333563
Cellfectin II	Thermo Fisher	Cat# 10458833
Geneticin (G418 sulfate)	Thermo Fisher	Cat# 10131-035
Gibco Grace’s Insect Medium, unsupplemented	Thermo Fisher	Cat# 11514546
Sf-900 II SFM	Thermo Fisher	Cat# 10902088
JF549	Promega	Cat# GA1110
Protein A Sepharose Cl-4B	Thermo Fisher	Cat# GE17-0963-02
Dynabeads Goat Anti-Mouse IgG	Thermo Fisher	Cat# 11033
Anti-FLAG M2 agarose resin	Sigma-Aldrich	Cat# A2220
Ni^2^–NTA agarose resin	QIAGEN	Cat# 30210
Trioxsalen (TMP)	Sigma-Aldrich	Cat# 6137
Mitomycin C from *Streptomyces caespitosus*	Sigma-Aldrich	Cat# M4287
Benzonase	Sigma-Aldrich	Cat# E1014
CK2	NEB	Cat# P6010S
Lambda protein phosphatase	NEB	Cat# P0753S
CX-4549	Enzo Life Sciences	Cat# ENZ-CHM151-0001
Propidium iodide	Sigma-Aldrich	Cat# P4170
Ribonuclease A	Sigma-Aldrich	Cat# R5503
Critical Commercial Assays		
DNA sequencing	Source BioScience	N/A

**Experimental models: Cell lines**		

HeLa FANCD2 −/−	Lopez-Martinez et al. ^10^	N/A
HeLa FANCD2 −/− + EGFP-FANCD2 + mCherry-UHRF1	Lopez-Martinez et al.^10^	N/A
HeLa FANCD2 −/− + EGFP-FANCD2-6A + mCherry-UHRF1	Lopez-Martinez et al.^10^	N/A
HeLa Halo-FANCD2 knock-in + EGFP-1-110-Geminin	Kupculak et al.^9^	N/A
HeLa + EGFP-FANCI + mCherry-UHRF1	This paper	N/A
HeLa PPP2R3A −/−	This paper	N/A
HeLa FANCD2 −/− PPP2R3A −/−	This paper	N/A

**Recombinant DNA**		

pOZ-N	Nakatani and Ogryzko^29^	N/A
pOZ-N-EGFP-FANCD2	This paper	N/A
pOZ-N-EGFP-FANCD2-6A	This paper	N/A
pOZ-N-EGFP-FANCI	This paper	N/A
pBlueScript II SK (+)	Addgene	Cat# 10359-016
pFB-FLAG-HA	Liang et al.^3^	N/A
pFastBac1	Thermo-Fisher	Cat# 10360014
pSpCas9(BB)-2A-Puro (PX459)	Addgene	Cat# 48139

**Oligonucleotides**		

siCtrl: 5′-UUCUCCGAACGUGUCACGUTT-3′	Eurofins	N/A
siPPP2CA (SMARTPool)	Horizon Discovery	Cat# L-003598-01-0020
siPPP2CB (SMARTPool)	Horizon Discovery	Cat# L-003599-00-0020
siPPP2R2A	Ambion Life Technology	Cat# S608 and Cat# S610
siPPP2R2B	Ambion Life Technology	Cat# S10969 and Cat# S10971
siPPP2R2C	Ambion Life Technology	Cat# S10972 and Cat# S10973
siPPP2R2D	Ambion Life Technology	Cat# S31640 and Cat# S224395
siPPP2R3A	Ambion Life Technology	Cat# S10976 and Cat# S10977
siPPP2R3B	Ambion Life Technology	Cat# S26253 and Cat# S26254
siPPP2R3C	Ambion Life Technology	Cat# S29986 and Cat# S29987
siPPP2R5C	Ambion Life Technology	Cat# S10987 and Cat# S10988
siPPP2R5D	Ambion Life Technology	Cat# S10990 and Cat# S10992
siPPP2R4 (PTPA)	Ambion Life Technology	Cat# S10978 and Cat# S10980
siSTRN3	Ambion Life Technology	Cat# S226265 and Cat# S226266
siPPP2R1A (SMARTpool)	Horizon Discovery	Cat# L-010259-00-0005
siPPP2R1B (SMARTpool)	Horizon Discovery	Cat# L-017592-00-0005

**Software and algorithms**		

Volocity	Quorum Technologies	N/A
Fiji	Fiji.sc	N/A
PyMOL	Pymol.org	N/A
MATLAB R2020b	Mathworks	N/A
Stormtrack	Uphoff et al.^30^	N/A
Graphpad prism 9	Graphpad by Dotmatics	N/A

**Other**		

PE Ultraview Spinning Disk Microscope	Micron Facility, University of Oxford	N/A
SPT microscope (PALM microscope)	Micron Facility, University of Oxford	N/A
Spectrolinker XL-1500 (365 nm)	Department of Biochemistry, University of Oxford	N/A
Cloning cylinders, glass	Sigma-Aldrich	Cat# C1059-1EA

### Experimental model and study participant details

#### Cell lines

HeLa and Phoenix A cell lines (originally from ATCC) were grown in Dulbecco’s modified eagle medium (D5796, Sigma Aldrich) supplemented with 10% Fetal Bovine Serum (F7524, Sigma Aldrich). Cells were incubated at 37°C and 5% CO_2_ and passaged using Trypsin-EDTA (0.25%, T4049, Sigma Aldrich).

### Method details

#### Plasmids and transfection

EGFP-fused proteins were expressed using the pOZ-N plasmid.^29^ The point mutations were gradually introduced by inverse PCR-based site-directed mutagenesis (Fisher and Pei, 1997) into pFB-EGFP-FANCD2-WT construct and the mutated FANCD2 was subsequently subcloned into the pOZ vector. The final constructs were introduced to cells by retroviral transfection using FuGENE6 (Promega) while following the manufacturer’s protocol.

siRNA transfection was performed as follows. A total number of 6 x 10^5^ HeLa cells are seeded in a 6-well plate a day before transfection. On the day of transfection, siRNAs are mixed with Lipofectamine RNAiMAX according to the manufacturer’s recommendations. Two siRNAs with different target sequences were used in combination for each gene at a final concentration of 25 nM for each, except for the two PP2A catalytic subunits for which SMARTpools were used at a final concentration of 25 nM. After three days of incubation with siRNAs at the 37°C incubator, cells were harvested for downstream experiments.

#### CRISPR/Cas9 gene editing

HeLa PPP2R3A −/− cells were generated using the pX459 plasmid (Addgene #48139). The guide sequence before the PAM sequence is 5′-TGTGAACCACTACAGCAGCG-3’. Primers (5′-CACCGTGTGAACCACTACAGCAGCG-3′ and 5′-AAACCGCTGCTGTAGTGGTTCACAC-3′) are annealed and phosphorylated before cloning into pX459 digested with BbsI. HeLa cells were electroporated with 5 μg of the resulting pX459-based plasmid. After 24 h of electroporation, cells were transiently selected by 2 μg/mL puromycin for 24 h then plated at a low density (100–300 cells) in 15-cm plates to grow single cell clones. After two weeks, single cell clones were isolated using 150 μL glass cloning cylinders and transferred to 24-well plates. Protein expression was examined by immunoblot analysis to confirm the depletion of PPP2R3A.

#### Clonogenic survival assay

Cells (400/800/2,000 for HeLa FANCD2^−/−^; 200/400/1,000 for all other cell lines) were plated in six-well plates and treated with different dosages of ICL inducing agents the next day. Colony formation was scored after 10–14 days of incubation by staining with 1% (w/v) crystal violet in methanol.

#### Cell cycle analysis (FACS)

HeLa cells were incubated with 20 ng/mL MMC for 2 h before recovering in DMEM for 24 h. Cells were harvested and washed with cold PBS containing 1 mM EDTA. Cells were collected and the concentration was adjusted to 2 x 10^6^ cells/mL, then 1 mL of the suspension was added dropwise into 9 mL of 70% cold ethanol and incubated for 18 h at 4°C. Samples were then centrifuged at 200 xg for 10 min. The pellet was then washed three times with cold PBS supplemented with 1 mM EDTA before staining with 20 μg/mL propidium iodide in PBS containing 1 mM EDTA, 0.1% Triton X-100 and 0.2 mg/mL RNase A at RT for 30 min. The cell cycle profile was assessed using a FACSCalibur flow cytometer and the data were analyzed by BD CellQuest.

#### Antibodies

Antibodies used were as follows: anti-FANCD2, 1:100 dilution (sc-20022, Santa Cruz Biotechnology); anti-FANCI, 1:500 dilution (FARF); anti-Flag, 1:1,000 dilution (M5, F4042, Sigma-Aldrich); anti-UHRF1, 1:1,000 dilution (sc-373750, Santa Cruz Biotechnology); anti-α-Tubulin, 1:2,000 dilution (05–829, Merck-Millipore).

#### Whole cell lysate preparation

Cells were scrapped off the dishes, collected into 1.5 mL Eppendorf tubes, spun down at 17,000xg for 5-10sec and washed with PBS. After aspirating the supernatants, the pellets were resuspended in an equal volume of benzonase buffer (2 mM MgCl_2_, 20 mM Tris pH 8.0, 10% glycerol, 1% Triton X-100 and 12.5 units/ml benzonase (E1014, Sigma)) and left on ice for 10–15 min. To lyse the cells, an equal volume of 2% SDS was subsequently added to each sample which was then heated at 70°C for 2 min. The protein concentration was determined using Bradford assay (Bio-Rad Life Science.).

#### Immunoblotting

Protein extracts were mixed with 6x SDS loading buffer (0.375 M Tris pH 6.8, 12% SDS, 60% Glycerol, 0.06% BPB) and supplemented with DTT to a final concentration of 50 mM DTT, topped up with milliQ to achieve a final concentration of 1 μg/μl and heated at 95°C for 5–10 min. The samples were loaded into an SDS-polyacrylamide gel along with the Mark 12 Protein Standard (Thermo Fisher Scientific) and run in 1x Running buffer for 2.5–3.5 h at 70-100V. The running buffer contained 25mM Tris, 192mM glycine and 0.1% SDS. Separating gel contained 5%–10% acrylamide/bis-acrylamide, 400 mM Tris HCl pH 8.8, 0.1% SDS, 0.1% APS (ammonium persulphate) and 0.1% TEMED (Sigma), stacking gel contained 5% acrylamide/bis-acrylamide, 125 mM Tris HCl pH 6.8, 0.1% SDS, 0.1% APS and 0.1% TEMED. After the run, proteins were transferred onto a nitrocellulose membrane (Amersham) by electroblotting, which was then stained with 1x Ponceau S (VWR Chemicals) dye, and the regions of interest were cut out. The membranes were blocked in 5% milk dissolved in 1x TBS-T (150 mM NaCl, 20 mM Tris pH 8.0, 0.1% Tween 20) for 1- 3h at the room temperature. Primary antibody incubations were carried out overnight at 4°C in 5% milk complemented with 0.1% sodium azide. On the following day, the membranes were washed three times for 5 min in 1x TBS-T and incubated with secondary antibodies for 1h at the room temperature. After 3 subsequent washes for 7 min in TBS-T the membranes were coated with ECL Western blotting Substrate (Perking Elmer) and developed on X-ray films (Amersham).

#### Protein purification

Purification of the FANCD2/FANCI complex, or its mutant derivatives, including FANCD2-6A/FANCI and FANCD2-6D/FANCI, and the proteins for the ubiquitination assay was the same as the previously reported method.^10^^,^^31^ Briefly, for FANCD2/FANCI complexes and UBA1, Sf9 cells were washed with PBS, harvested, resuspended in lysis buffer (20 mM Tris-HCl pH 8.0, 0.1 M KCl, 10% glycerol, 0.2 mM PMSF, 2 mM β-mercaptoethanol) followed by sonication (4 × 10 s bursts on ice). Lysates were clarified by 20 min centrifugation (17,000g), and the collected supernatants were incubated with M2 anti-FLAG agarose resin (A2220, Sigma) at 4°C for 2h. The resin was extensively washed in the original lysis buffer and the proteins were eluted in the lysis buffer complemented with 0.5 mg/mL FLAG peptide. For FANCL, Sf9 cells were washed, harvested and resuspended in detergent-containing lysis buffer (20 mM Tris-HCl pH 8.0, 0.1 M KCl, 10% glycerol, 0.2 mM PMSF and 2 mM β-mercaptoethanol, 0.1% Tween 20) without any sonication. The rest of the purification proceeded the same way as for FANCD2/FANCI complexes and UBA1. 6xHis Ubiquitin was expressed in E. coli BL21(DE3) (Novagen). The cells were grown in Lysogeny Broth supplemented with antibiotics at 37°C until OD600 0.6–0.8 was reached. The expression of 6xHis ubiquitin was then induced by 0.5 mM IPTG and the cells were left to grow overnight at 16°C. Harvested cells were lysed using a french press in a buffer containing 0.5 M NaCl, 0.1 M Tris pH 8, 0.02 M Imidazole and 0.25 mM TCEP. The lysate was then clarified by centrifugation (48,000g) and the supernatant was incubated with Ni^2+^-NTA agarose (QIAGEN) for 2 h at 4°C. Finally, ubiquitin was loaded onto a Superdex 200 column with 200 mM NaCl, 0.1 M Tris (pH 8.0), 10% glycerol and after the elution was finished, the indicated fractions collected and concentrated using VivaSpin 20 (Sartorius).

The PPP2R3A/PP2A holoenzyme was purified by employing the MultiBac expression system. Flag-PPP2CA, His-PPP2R1A and HA-PPP2R3A were cloned into the pACEBac1 as a combined vector and transformed into DH10Bac competent *E. coli* cells for generation of the bacmid. Specifically, DH10Bac competent cells were thawed on ice and incubated with 500 ng purified pACEBac1 plasmid at 4°C for 25 min, followed by heat shock for 40 s at 42°C. Cells were immediately transferred back on ice to cool down for 2 min and were subsequently recovered by shaking with 1 mL LB medium at 37 °C at 225 rpm for 4 h. After that, the medium was removed by centrifugation at 17,000 x g for 2 min, and the pellet was resuspended and plated on an agar plate containing 50 μg/mL kanamycin, 7 μg/mL gentamicin, 10 μg/mL tetracycline, 100 μg/mL Bluo-gal, and 40 μg/mL IPTG. The plate was incubated in a 37°C incubator overnight, positive colonies were selected by blue/white selection. The positive bacmid was then transfected into Sf9 cells to produce baculovirus. 1 μg bacmid was transfected into 5 × 10^5^ Sf9 cells by mixing the bacmid with 2 mL Grace’s Insect Cell Culture Medium and 6 μL Cellfectin II. Cells were incubated at 27°C for 5 hours before the medium was replaced with 2 mL Sf-900 II SFM medium. Five days post transfection, the baculovirus was harvested by collecting the 2 mL culture medium. The virus was passaged twice before being used for the transduction of Sf9 cells. 200 μL virus (the value depended on the viral titer) was added to 15 million Sf9 cells in 20 mL Sf-900 II SFM medium in a 15 cm dish. Cells were harvested three days after infection by scraping with lysis buffer (20 mM Tris-HCl pH 8.0, 100mM KCl, 10% glycerol, 2 mM β-mercaptoethanol, and 0.2 mM PMSF) followed by sonication (4 × 10s bursts on ice). After centrifugation at 17,000 x g for 5 min, the supernatants were incubated with 50 μL M2 anti-Flag agarose resin for 2 h at 4°C. After extensive washes, the proteins were eluted from the resin in 0.5 mg/mL Flag peptide in the same lysis buffer. The eluate was firstly run on a Superdex 200 26/60 column (GE Healthcare Life Sciences) in 20 mM Tris-HCl pH 8.0, 100 mM KCl, 10% glycerol and 2 mM β-mercaptoethanol and 0.2 mM PMSF. Fractions containing the complex were combined and subsequently passed through a Mono Q anion exchange chromatography column (GE Healthcare Life Sciences) to further remove impurities. Proteins were eluted by a salt gradient of KCl from 10 mM to 1 M. Finally, eluates were combined and concentrated by PEG for subsequent *in vitro* assays.

#### *In vitro* phosphorylation and dephosphorylation assays

Phosphorylation of the FANCD2/FANCI complex was done by mixing FANCD2/FANCI with either CK2 (P6010, NEB) or purified ATR at a final concentration of 7.32 nM and 131 nM, respectively, and incubated at 30°C for 30 min in 50 mM Tris pH 7.5, 10 mM MgCl_2_, 2 mM DTT, 0.1 mM EDTA and 2 mM ATP. The dephosphorylation reaction was carried out by mixing FANCD2/FANCI with the purified PPP2CA/PPP2R1A/PPP2R3A complex at a final concentration of 3.2 nM, followed by incubation at 30°C for 60 min in 50 mM HEPES pH 7.5, 100 mM NaCl, 2 mM DTT, 1 mM MnCl_2_ and 0.5 μM CX-4549 (CK2 inhibitor).

#### *In vitro* monoubiquitination assay

The described *in vitro* monoubiquitination assay is a derivative of a previously published method.^32^ Reaction volumes of 25 μL contained 17 nM UBA1, 0.64 mM UBE2T, 4.2 mM 6xHis-Ub, 0.37 mM FANCL, 0.25 mM FANCD2/FANCI or its mutants and 20 mM pBlueScript SKII(+) in the following reaction buffer: 50 mM Tris (pH 7.5), 100 mM KCl_2_, 2 mM MgCl_2_, 0.5 mM DTT and 2 mM ATP. Reactions were vortexed and incubated at room temperature for indicated times, negative control reactions were incubated for the longest indicated time but contained no ATP. To terminate the reactions, 6x SDS loading buffer and 10x Reducing agent were used. The samples were loaded on a 5% SDS-PAA gel and subjected to Coomassie brilliant blue staining.

#### Live-cell imaging using confocal microscopy

Live cell imaging experiments were carried out using an OLYMPUS IX81 microscope connected to a PerkinElmer UltraView Vox spinning disk system equipped with a Plan-Apochromat 60x/1.4 oil objective, the software in use for image capturing was Volocity software 6.3. The stable temperature and CO_2_ concentration during the recordings was maintained using a live cell environmental chamber (Tokai hit). The excitations for EGFP and mCherry were 488nm and 561nm, respectively. Confocal image series were recorded with a frame size of 512 × 512 pixels and a pixel size of 139 nm. For localized introduction of interstrand crosslinks, cells were plated onto glass bottom dishes (MatTek) and incubated with 20 μg/mL 4,5′,8-trimethylpsoralen (TMP) for 30–60 min at 37°C. Subsequent microirradiation was performed using the FRAP preview mode of the Volocity software by scanning (100 ms for each irradiation time) a preselected area within the nucleus with 65 cycles of the 405nm laser which was set to 1.12 mW per cycle. The intensities of both mCherry and EGFP at microirradiated sites were quantified using ImageJ or Fiji and normalized by their intensities before microirradiation essentially as previously described^33^ as follows. The images were acquired in the green (488 nm) and/or red (561 nm) fluorescence channels. The camera used for acquisition was Hamamatsu C9100-13. All images were acquired and processed using the Volocity software. After acquisition the images were analyzed with ImageJ or Fiji software where the following measurements were taken. First, the areas where the cells were irradiated were marked by hand using the free-select tool. It was necessary to do this by hand for each image because the irradiated areas sometimes move slightly during long acquisitions due to cell migration. Second, measurement of the brightness of the selected field was performed and recorded. Third, the rest of the cell nucleus was selected avoiding the irradiated area, and the same measurement was performed. Fourth, the background was selected and its intensity measured. Then, for each cell, the following formula was used: Sx=(Ax-B)/(Cx-B), where Ax represents the brightness of the stripe, Cx represents the brightness of the rest of the rest of the cell nucleus, and B represents the brightness of the background in given field of view. The formula is returning Sx, which represents the relative brightness of the stripe compared to the rest of the nucleus in the given cell. After obtaining Sx values for each of the cells in the view field and for each time point of interest, the average S value was calculated along with the standard error (here standard deviation divided by the square root of the number of all the cells taken into consideration in the given view field). To make the comparison between the experiments easier, measurements were then normalized in such way that the first time point (before the irradiation) would always equal 1. To normalize, the difference between the first timepoint and 1 was added or subtracted in all later measurements (so for example if the average value for all cells in the first timepoint was giving the value of 1.12, 0.12 would be subtracted from this and all future timepoints). It was a necessary adjustment since, the distribution of the proteins observed in our experiments is not uniform in the nucleus, with dark areas and sometimes bright foci. Since we usually tried to locate the irradiation area in such a way to avoid irradiating these non-uniform areas, its brightness would not exactly be equal to that of the rest of the nucleus even in the first time point before the irradiation.

#### Live-cell single-molecule tracking analysis

Cells were plated on gelatinised 35mm glass-bottom petri dishes (MatTek) at least 12 h before imaging. Cells were labeled in 5 nM Jenelia Fluor 549 (JF-549) for 30–60 min and washed twice with PBS and one time with colorless DMEM (D7777, Sigma Aldrich) supplemented with 10% FBS, 2mM L-glycine (G7513, Sigma Aldrich) for 5 min in a 5% CO_2_ incubator. 20 mM HEPES pH 7.5 were supplemented to the medium before imaging. Single-molecule tracking experiments were conducted on a custom-built PALM/TIRF (Photoactivated localization microscopy/Total internal reflection fluorescence) microscope with a highly inclined and laminated optical sheet (HILO) microscopy setting.^30^ The best HILO angle was found before each experiment by maximizing the brightness of the fluorescent spots in the chosen focal plane. The sample temperature during the imaging was maintained at 37°C by using a heated stage. For determining different cell phases, the absence or presence of EGFP-1-110-Geminin in nuclei was empirically evaluated by inspecting cells using 0.5% 488 nm excitation laser with a GFP channel filter. For tracking of diffusing Halo-FANCD2 molecules, movies were recorded with continuous ∼10% 561 nm laser excitation. To introduce ICL damage in cells, 20% 405 nm laser illumination was used to treat cells for 20 s and imaging was performed post-irradiation after 15 min incubation. Movies of 2,000–4,000 frames were acquired at 20 ms per frame using a 256 × 256 pixels (0.096 μm/pixel) region of interest. The obtained movies were quantified in MATLAB.^30^ Further data processing and statistical analysis were performed using Excel and GraphPad Prism 8. Positions of fluorescent molecules were detected in each frame using thresholding of band-pass filtered images, followed by localization using a phasor algorithm.^34^ Localizations were linked to tracks if they remain within a circular region of 8 pixels radius in consecutive frames. A tracking memory parameter of 1 frame allowed for blinking or missed detection of a molecule. The mean diffusion coefficient (D) for each track was calculated from the mean squared displacement (MSD): D = MSD/(4⋅Δt), with Δt = 20 m. Tracks with less than 4 steps were discarded from the analysis. The population of bound molecules was quantified based on the fraction of tracks with D ≤ 0.3 μm^2^/s.^9^

### Quantification and statistical analysis

Statistical parameters, including statistical tests used, number of events quantified, standard error of the mean, and statistical significance are reported in the figures and in the figure legends. Statistical analysis has been performed using Microsoft Office Excel software and statistical significance is determined by the value of *p* < 0.05.

## Supplemental information

Document S1. Figures S1–S5

Document S2. Article plus supplemental information
