# Supplementary material for: PP2A licenses the FANCD2/FANCI complex for chromosome loading
Source: Cell Rep. 2024 Nov 12;43(11):114971. doi: 10.1016/j.celrep.2024.114971 (PMC12979246; doi:10.1016/j.celrep.2024.114971)
Supplement: Document S1. Figures S1–S5 [file mmc1.pdf]

Cell Reports, Volume *43*

Supplemental information

PP2A licenses the FANCD2/FANCI complex for chromosome loading

Di Yang, Fengxiang Bai, David Lopez Martinez, Hannan Xu, Ai Johjima-Murata, Lily Jiaqi Cao, and Martin A. Cohn

Figure S1

A

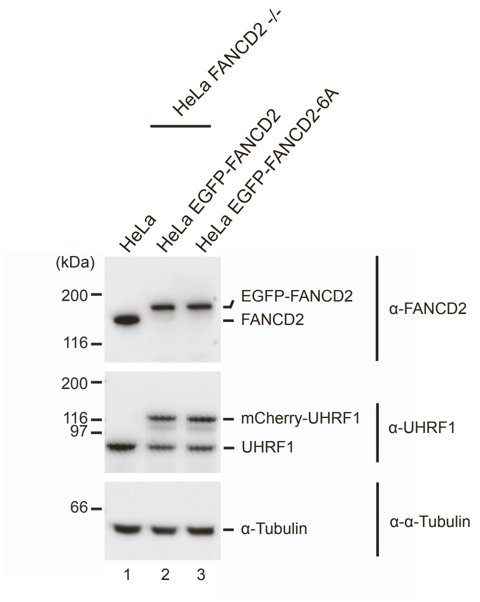

B

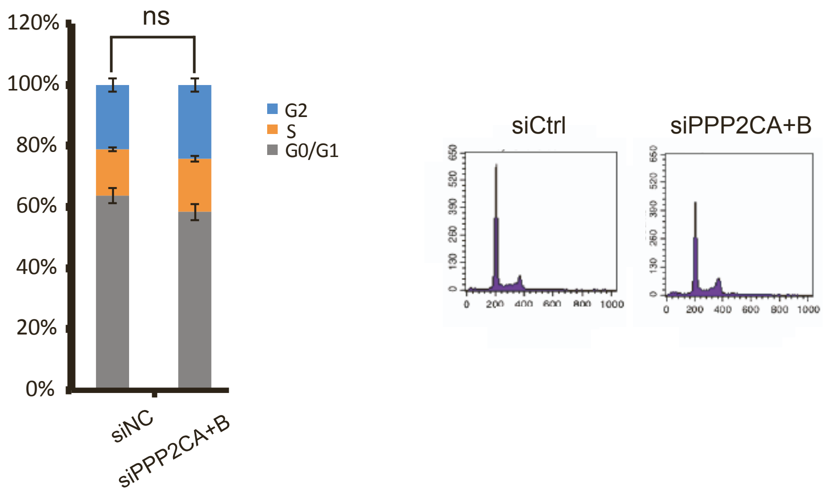

C

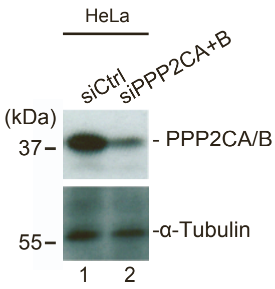

Figure S1 (data relating to Figure 1). Expression levels of proteins expressed exogenously in HeLa cells. A) Immunoblot analysis of HeLa cells stably expressing EGFP-FANCD2 and mCherry-UHRF1. B) Cell cycle profile measured by DNA content of HeLa cells with PP2A catalytic subunits depleted, and cells treated with 20 ng/mL MMC for 2 h followed by 24 h recovery. FACS analysis. Quantification is shown in the histogram at the left. C) Immunoblot analysis showing the knock-down of PP2A catalytic subunits in cells used in (B).

Figure S2

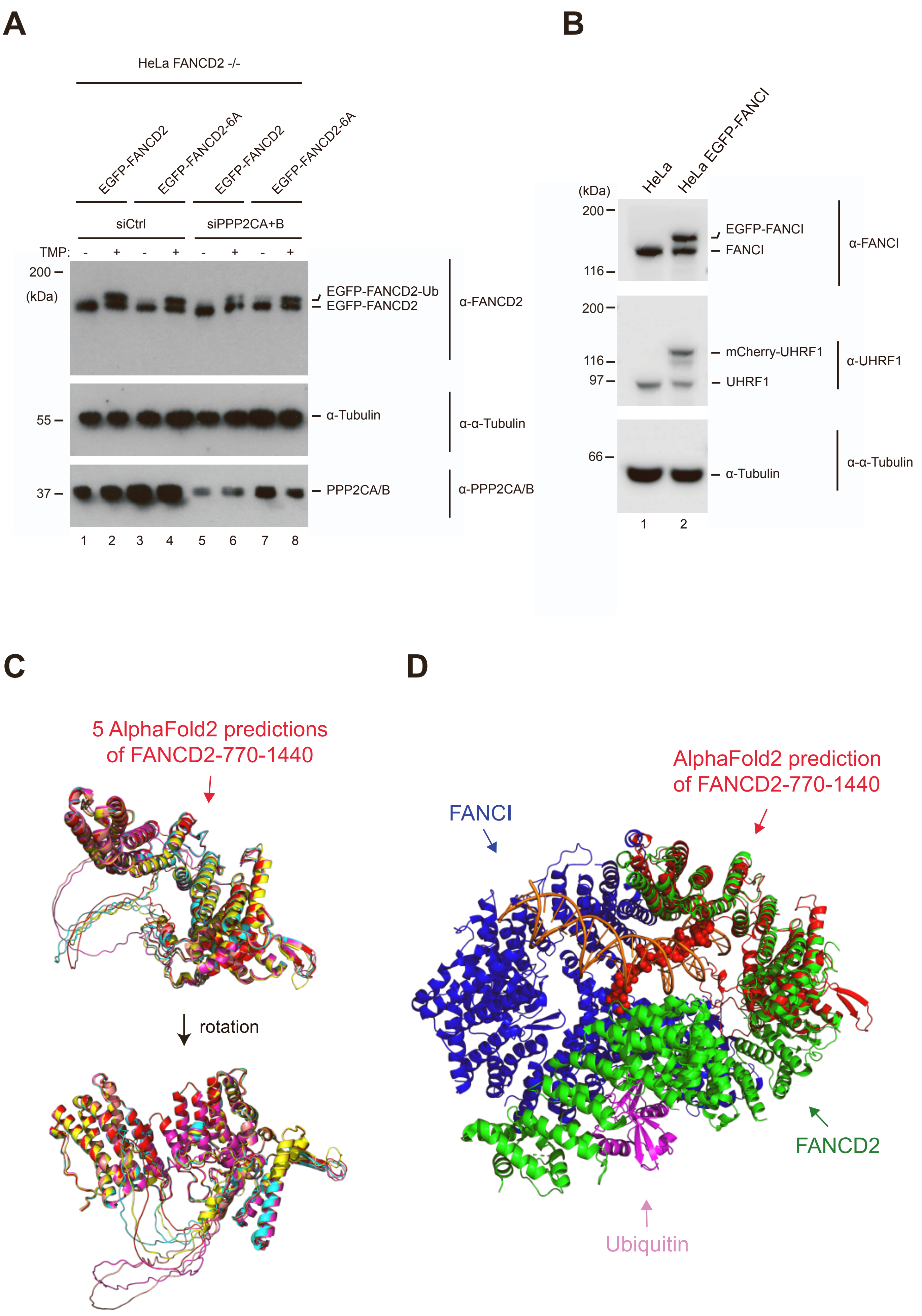
A
HeLa FANCD2 -/-
EGFP-FANCD2
EGFP-FANCD2-6A
EGFP-FANCD2
EGFP-FANCD2-6A
siCtrl
siPPP2CA+B
TMP: - + - + - + - +
200
(kDa)
EGFP-FANCD2-Ub
EGFP-FANCD2
α-FANCD2
55
α-Tubulin
α-α-Tubulin
37
PPP2CA/B
α-PPP2CA/B
1 2 3 4 5 6 7 8
B
HeLa
HeLa EGFP-FANCI
(kDa)
200
116
EGFP-FANCI
FANCI
α-FANCI
200
116
97
mCherry-UHRF1
UHRF1
α-UHRF1
66
α-Tubulin
α-α-Tubulin
1 2
C
5 AlphaFold2 predictions
of FANCD2-770-1440
rotation
D
AlphaFold2 prediction
of FANCD2-770-1440
FANCI
FANCD2
Ubiquitin

Figure S2 (data relating to Figure 2). Expression of exogenous proteins in HeLa cells. A) Immunoblot analysis of cells lines used for live-cell imaging in Figure 2E. The blot shows FANCD2 monoubiquitination in HeLa FANCD2 -/- cells complemented with either EGFP-FANCD2 or EGFP-FANCD2-6A transfected by siRNAs targeting the PP2A catalytic subunits. B) Immunoblot analysis of cell line used for live-cell imaging in Figure 2B. C) AlphaFold2 structural prediction of amino acids 770-1400 of FANCD2. 5 predictions are shown. The prediction shown in red color is the same prediction that is also shown in (D). D) Cryo-EM structure of ubiquitinated FANCD2/FANCI complex (PDB: 6VAE) bound to DNA (Wang et al., 2020). FANCD2 is shown in green, FANCI in blue, and ubiquitin in pink. AlphaFold2 was used to predict the structure of FANCD2-770-1400, shown in red and superimposed onto the cryo-EM structure. Amino acids 880-898 containing the PP2A cluster, are shown as spheres to enhance visibility. The AlphaFold2 prediction shown is the same as the one shown in red color in (C).

Figure S3

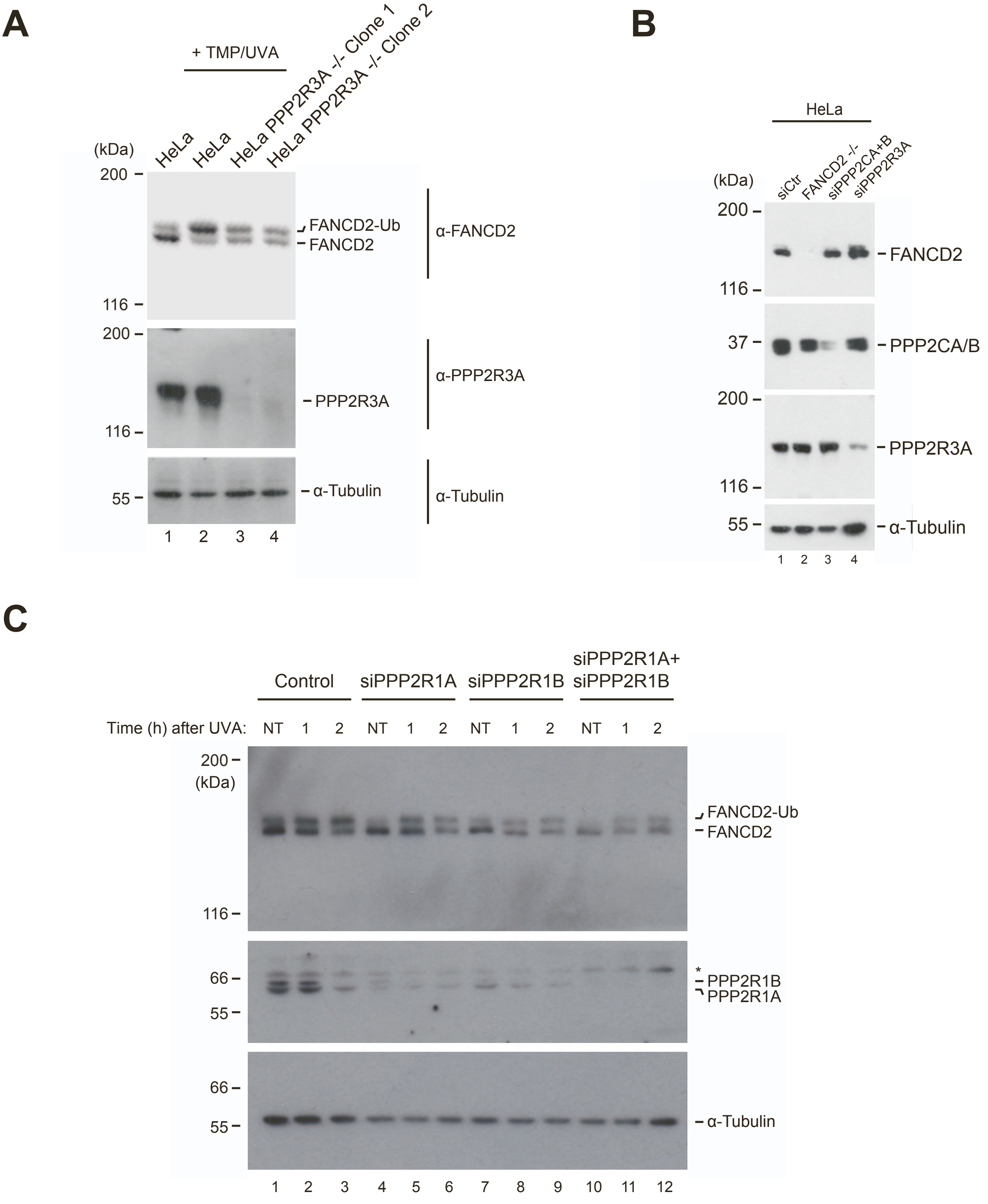

Figure S3 (data relating to Figure 3). Suppression of PP2A leads to reduced monoubiquitination of FANCD2. A) Immunoblot analysis of HeLa PPP2R3A -/- cell lines used in Figure 3D. B) Immunoblot analysis showing the knock-down of relevant genes by siRNAs in cells lines used for FACS analysis in Figure 3F. C) Immunoblot analysis of HeLa cells transfected with siRNAs targeting PPP2R1A and PPP2R1B, followed by TMP/UVA treatment. Cells were harvested 0-2 h post treatment.

Figure S4

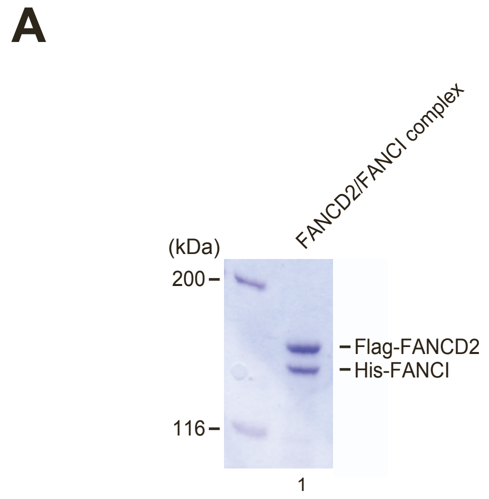

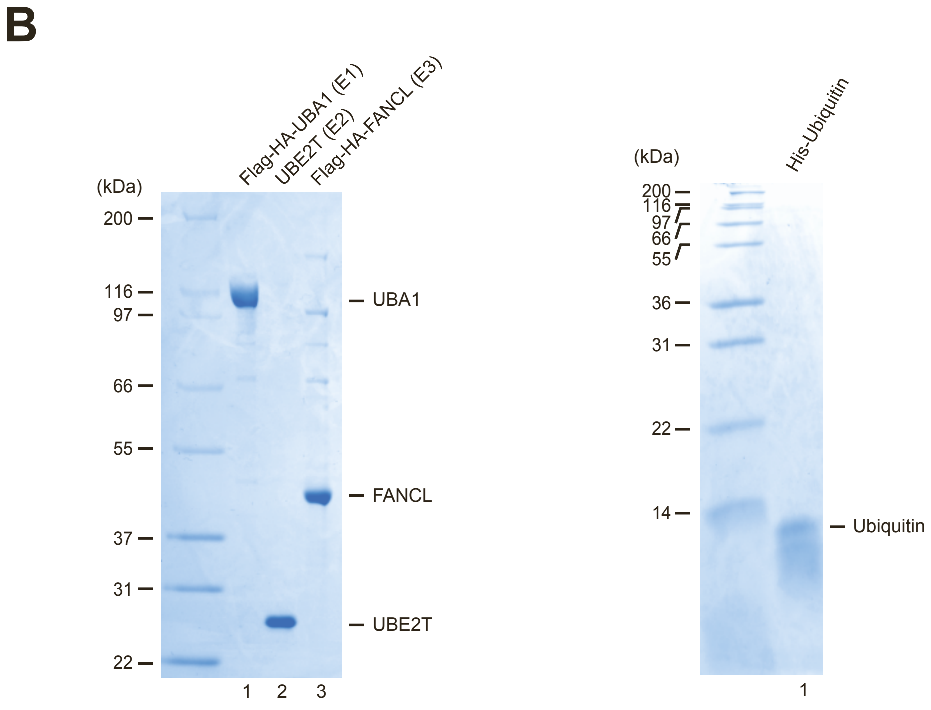

Figure S4 (data relating to Figure 5). Purification of recombinant proteins. A) Coomassie brilliant blue stain of recombinant FANCD2/FANCI complex used in the *in vitro* phosphorylation/dephosphorylation assays in Figure 5B-C. B) Coomassie brilliant blue stain of recombinant proteins used in the *in vitro* ubiquitination assay in Figure 5D.

Figure S5

A

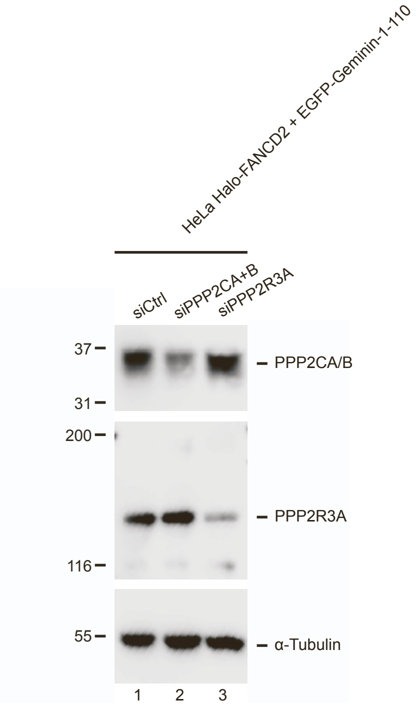

Figure S5 (data relating to Figure 6). Depletion of PP2A subunits in HeLa cells. (A) Immunoblot analysis showing the knock-down of relevant genes by siRNAs in cell lines used in Figure 6E.
